# Viscosity-dependent determinants of *Campylobacter jejuni* impacting the velocity of flagellar motility

**DOI:** 10.1128/mbio.02544-23

**Published:** 2023-12-12

**Authors:** Deborah A. Ribardo, Jeremiah J. Johnson, David R. Hendrixson

**Affiliations:** 1 Department of Microbiology, University of Texas Southwestern Medical Center, Dallas, Texas, USA; 2 Department of Microbiology, University of Tennessee, Knoxville, Tennessee, USA; Fred Hutchinson Cancer Center, Seattle, Washington, USA

**Keywords:** flagellar motility, viscosity, swimming velocity, *Campylobacter jejuni*

## Abstract

*Campylobacter jejuni* colonizes the thick mucus layer lining the lower intestinal epithelium of hosts to promote commensalism in birds and animals or diarrheal disease in humans. *C. jejuni* displays higher swimming velocity as external viscosity increases, which likely benefits colonization and persistence in the intestinal mucus layer of hosts. *C. jejuni* produces one of the most structurally complex bacterial flagellar motors, which has been proposed to generate high torque for high swimming velocity. However, it is unknown how this flagellar motor alters output to impact swimming velocities in a viscosity-dependent manner. In this work, we identified viscosity-dependent determinant A (VidA) (*Cjj81176_0996*) and VidB (*Cjj81176_1107*) in modulating swimming velocity primarily in low-viscosity environments. *C. jejuni* Δ*vidA* cells were non-motile or swam slowly in low-viscosity media, but swam with high velocity similar to wild-type cells in both Newtonian and non-Newtonian media with high viscosity. These data indicate that VidA is required for swimming in low-viscosity environments. Suppressor mutants suggested that the lower swimming velocity of Δ*vidA* in low viscosity was due to unregulated activity of VidB, which we propose has a brake- or clutch-like activity to reduce swimming velocity in low viscosity. Unlike other bacterial flagellar brakes or clutches, we found no evidence for cyclic diguanylate monophosphate influencing VidB activity. Our analyses suggest that the mechanics of the *C. jejuni* flagellar motor has naturally evolved for high-velocity swimming in milieus with high viscosity and requires the unique combination of VidA and VidB to modulate its activity to slow swimming velocity in low-viscosity environments.

**IMPORTANCE:**

Bacteria can adapt flagellar motor output in response to the load that the extracellular milieu imparts on the flagellar filament to enable propulsion. Bacteria can adapt flagellar motor output in response to the load that the extracellular milieu imparts on the flagellar filament to enable propulsion through diverse environments. These changes may involve increasing power and torque in high-viscosity environments or reducing power and flagellar rotation upon contact with a surface. *C. jejuni* swimming velocity in low-viscosity environments is comparable to other bacterial flagellates and increases significantly as external viscosity increases. In this work, we provide evidence that the mechanics of the *C. jejuni* flagellar motor has evolved to naturally promote high swimming velocity in high-viscosity environments. We found that *C. jejuni* produces VidA and VidB as auxiliary proteins to specifically affect flagellar motor activity in low viscosity to reduce swimming velocity. Our findings provide some of the first insights into different mechanisms that exist in bacteria to alter the mechanics of a flagellar motor, depending on the viscosity of extracellular environments.

## INTRODUCTION

The bacterial flagellum is a reversible rotary motor for swimming motility and navigation of individual cells through environments (1). The flagellum is composed of many proteins that form three main substructures: a basal body associated with the cytoplasmic membrane and connected to the periplasmic rod, a surface hook attached to the tip of the rod that serves as a universal joint, and an extracellular filament tethered to the hook that rotates to propel bacteria (2–4). The basal body contains core components of a flagellar type III secretion system (fT3SS) that secretes most proteins forming the flagellar structure (5–10). The fT3SS is surrounded by the membrane/supramembrane (MS) ring (a multimer of FliF) in the cytoplasmic membrane (11, 12). The C ring is associated with the cytoplasmic face of the MS ring and contains the rotor (a multimer of FliG), with the switch complex (formed by FliM, FliN, and/or FliY) that alters direction of the rotor and, hence, the flagellum (13–20). FliH attaches to the lower face of the C ring and serves as spokes to integrate the Fli_6_FliJ complex below the fT3SS to energize its secretory activity (21–25).

Rotation of the flagellar motor is powered by stator units in the cytoplasmic membrane that form a stator ring decorating the circumference of the rotor (26–33). A stator unit is composed of five MotA proteins and two MotB proteins (34, 35). MotA interacts with FliG subunits in the rotor, whereas MotB interacts with cell wall components (26, 36–38). Transport of protons or ions by the stators causes a conformation change in MotA to exert a pushing force on the rotor that generates torque to rotate the flagellum (26, 27, 39–43).

A small number of studies over the last few decades have measured swimming velocities for peritrichous and polar flagellates in both Newtonian and non-Newtonian fluids and estimated or calculate torque generated by the respective flagellar motors (44–48). Whereas water is a Newtonian fluid in which viscosity remains constant when stress is applied, the viscosity of non-Newtonian fluids such as ketchup or quicksand increases or decreases when a force or stress is applied. Although the bacterial species analyzed and viscosity agents employed have not been uniform in these studies, a relatively consistent observation is that the swimming velocity of bacterial flagellates increases to varying levels in non-Newtonian fluids as viscosity increases, but velocities decrease as viscosity increases in Newtonian fluids (44–47).

In comparison to most peritrichous organisms, polar flagellates generally possess higher swimming velocities (45, 48–51). For instance, *Campylobacter jejuni*, an amphitrichous polar flagellate that produces a single flagellum at each pole, has a similar swimming velocity at low viscosity as *Salmonella* and *Escherichia coli*, but swimming velocity increases to a greater extent than *Salmonella* and *E. coli* (50–100 μm/s for *C. jejuni* vs ~35 to 45 µm/s for *Salmonella* or *E. coli*) as viscosity in a non-Newtonian fluid increases (49, 50). The flagellar motors of *C. jejuni* and other polar flagellates, along with *Borrelia burgdorferi*, which produces polar periplasmic flagella, have been proposed to generate more torque to enable propulsion through viscous milieus, along with higher swimming velocities relative to peritrichous bacteria (49, 51–56).

The amount of torque generated by a flagellar motor to turn the rotor is influenced by the number of stator units incorporated into the motor and the distance the stators are positioned relative to the flagellar rod, the central axle of the motor (57–61). In some species, stator units are dynamic components, with a direct correlation between the number of stators per motor and the extracellular mechanical load upon the filament. In *E. coli*, one stator unit is sufficient to turn the flagellar motor, but the motor accommodates up to 11 stators as load increases (58, 59, 61, 62). As the external load on the filament increases, such as what occurs upon contact with a surface or in environments with higher viscosity, an increase in stator number increases the amount of power for the motor and torque generated to maintain propulsion of bacterial cells. Additionally, interactions of stator units with cell wall components have been observed to be strengthened by a catch-bond mechanism to retain stators within the motor for a longer period of time to generate more torque (63).

The flagellar motors of *B. burgdorferi* and many polar flagellates like *C. jejuni*, *Helicobacter pylori*, and *Vibrio* species have evolved wider rotors that enable an increase in stator unit occupancy (52, 56, 64–67). For example, *B. burgdorferi* incorporates 16 stator units around a 56-nm rotor, and *C. jejuni* incorporates 17 stator units around a 53-nm rotor, in contrast to *E. coli* and *Salmonella* species with up to 11 stator units surrounding a 44-nm rotor (52, 56, 64). This rotor enlargement coincides with the evolution of structural disks or scaffolds that position stators around the rotor at an increased distance from the flagellar rod (52, 54, 55, 64, 65, 68–75). These scaffolding structures of *B. burgdorferi* and *C. jejuni* flagellar motors may continuously allow maximal stator occupancy. Thus, stator number may be independent of load and not fluctuate in these motors. Both the increase in number and wider placement of the stators presumably increase the amount of torque generated by these motors that likely translates into increased motor output and the higher swimming velocities observed especially in viscous environments (48–50).

Flagella and flagellar motility are required by *C. jejuni* to infect the human lower intestinal tract to promote diarrheal disease and colonize the lower intestinal tract of avian species, such as chickens, and mammals for commensalism (76–79). *C. jejuni* efficiently colonizes the mucus layer atop the epithelia of these hosts. Intestinal mucus and mucus lining the epithelium in other tissues of the host are considered to behave as a non-Newtonian medium (80). In intestinal mucus, *C. jejuni* encounters low viscosity at the luminal surface of the mucus layer and higher viscosity as it moves deeper in the mucus toward the epithelial surface (79, 81–84). Thus, *C. jejuni* has evolved to swim efficiently and with high velocity in such a viscous environment to benefit the bacterium in colonizing human, avian, and other hosts. While stator number and a wider rotor may provide an explanation for how the *C. jejuni* flagellar motor generates more torque for high swimming velocities in viscous milieus relative to many other motile bacteria, other factors that may influence the motor to modulate swimming velocities in a viscosity-dependent manner are unknown. Recently, external viscosity was shown to facilitate the correct wrapping of leading and lagging polar filaments of *C. jejuni* to promote effective swimming at higher viscosities (85).

In this report, we identify two proteins of *C. jejuni*—viscosity-dependent determinant A (VidA) and VidB—that are responsible for modulating swimming velocity. However, these proteins primarily impact swimming velocity at low viscosity, with less influence on swimming velocity in high-viscosity Newtonian and non-Newtonian fluids. Further analysis suggested that VidB acts as a potential brake or clutch, while VidA represses this activity. As these auxiliary proteins appear to function at low viscosity, our findings suggest that the *C. jejuni* flagellar motor has evolved to power flagellar rotation and generate torque for high-velocity swimming in viscous environments by default. Our analysis of these proteins provide new insights into a different mechanism for how a high-torque flagellar motor can be altered to mediate different velocities of motility in a range of viscosities.

## RESULTS

### Characterization of swimming velocities of *C. jejuni* populations across viscosities in non-Newtonian fluids

**Fig 1:**
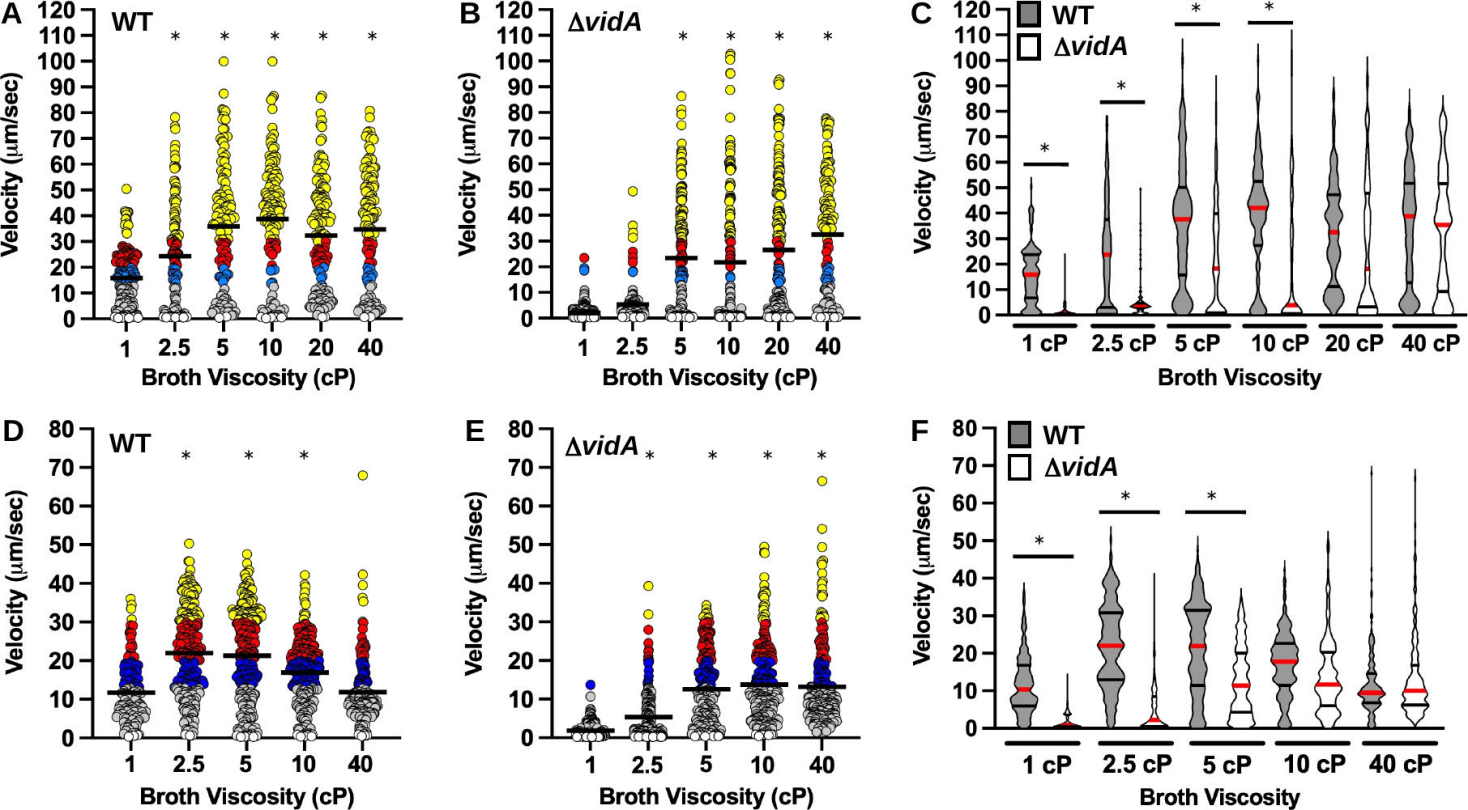
Swimming velocity of WT *C. jejuni* and *C. jejuni* Δ*vidA* in media with different viscosities. Swimming velocity of WT *C. jejuni* or *C. jejuni* Δ*vidA* populations after growth for 24 h in MH broth alone (1 cP) or MH broth with increasing concentrations of (**A–C**) methylcellulose or (**D–F**) Ficoll to increase extracellular viscosity up to 40 cP. Swimming velocities of individual cells (*n* > 100) were measured by video tracking under dark-field microscopy. Assays were performed in triplicate and combined. For panels **A B**, **D**, and (**E)**, each bar represents the mean swimming velocity of a population. Circles represent individual cells with velocities of < µm/s (white), 1–13 μm/s (gray), 13–20 μm/s (blue), 20–30 μm/s (red), and >30 µm/s (yellow). Statistical significance of difference in swimming velocities between WT or Δ*vidA* at 1 cP to cells grown in media at other viscosities was calculated by one-way analysis of variance (ANOVA) followed by Tukey’s multiple comparisons test. *P < 0.05. (**C and F**) Violin plots of (**C**) the data presented in panels **A** and **B **and (**F**) the data presented in panels **D and E**. The red bar represents the median, and black bars represent the 25th and 75th quartiles. Statistical significance of difference in swimming velocities between WT (gray) and Δ*vidA* (white) cells in cultures grown at each viscosity was calculated by one-way ANOVA followed by Tukey’s multiple comparisons test. *P < 0.05.

We initially characterized the swimming velocity of wild-type (WT) *C. jejuni* strain 81–176 in Mueller-Hinton (MH) broth with physiologically relevant viscosities that the bacterium likely encounters in the intestinal lumen and mucus layer atop the intestinal epithelium of hosts. This *C. jejuni* strain is a clinical isolate from a patient with diarrheal disease that can also colonize the intestinal tract of avian hosts without causing disease (76, 79). Considering that in nature *C. jejuni* is found in the mucus layer atop the intestines of hosts and mucus has non-Newtonian properties (80), we initially chose to supplement MH broth with methylcellulose, a branched-chain polymer, to increase viscosity and create a non-Newtonian medium to analyze swimming velocities. *C. jejuni* was grown overnight in standing cultures of MH alone [representing low viscosity at 1 centipoise (cP)] or with increasing concentrations of methylcellulose to increase viscosity from 2.5 to 40 cP (the latter the approximate viscosity of corn oil at room temperature) in microaerobic conditions at 37°C. After growth, the motility tracks of over 100 individual *C. jejuni* cells were recorded by dark-field microscopy and then converted to swimming velocities. In MH broth alone (1 cP), the swimming velocity of individual WT cells ranged from 0.4 to 50.5 µm/s with a mean velocity of 15.9 µm/s (Fig. 1A and C). As viscosity increased, we observed both an increase in the mean swimming velocity and a wider range of velocities (Fig. 1A and C; Movies S1 and S2). The mean swimming velocity increased to 24.4 µm/s at 2.5 cP and reached an average between 32.3 and 38.8 μm/s at viscosities of 5–40 cP. In these higher viscosities, the majority of cells were swimming higher than 30 µm/s. These data suggest that the *C. jejuni* flagellar motor promotes a higher swimming velocity as the viscosity of a non-Newtoian fluid increases. This observation is similar to *E. coli*, which can swim with higher velocity to an extent as the viscosity of a non-Newtonian fluid increases with increasing methylcellulose concentrations (46).

**Fig 2:**
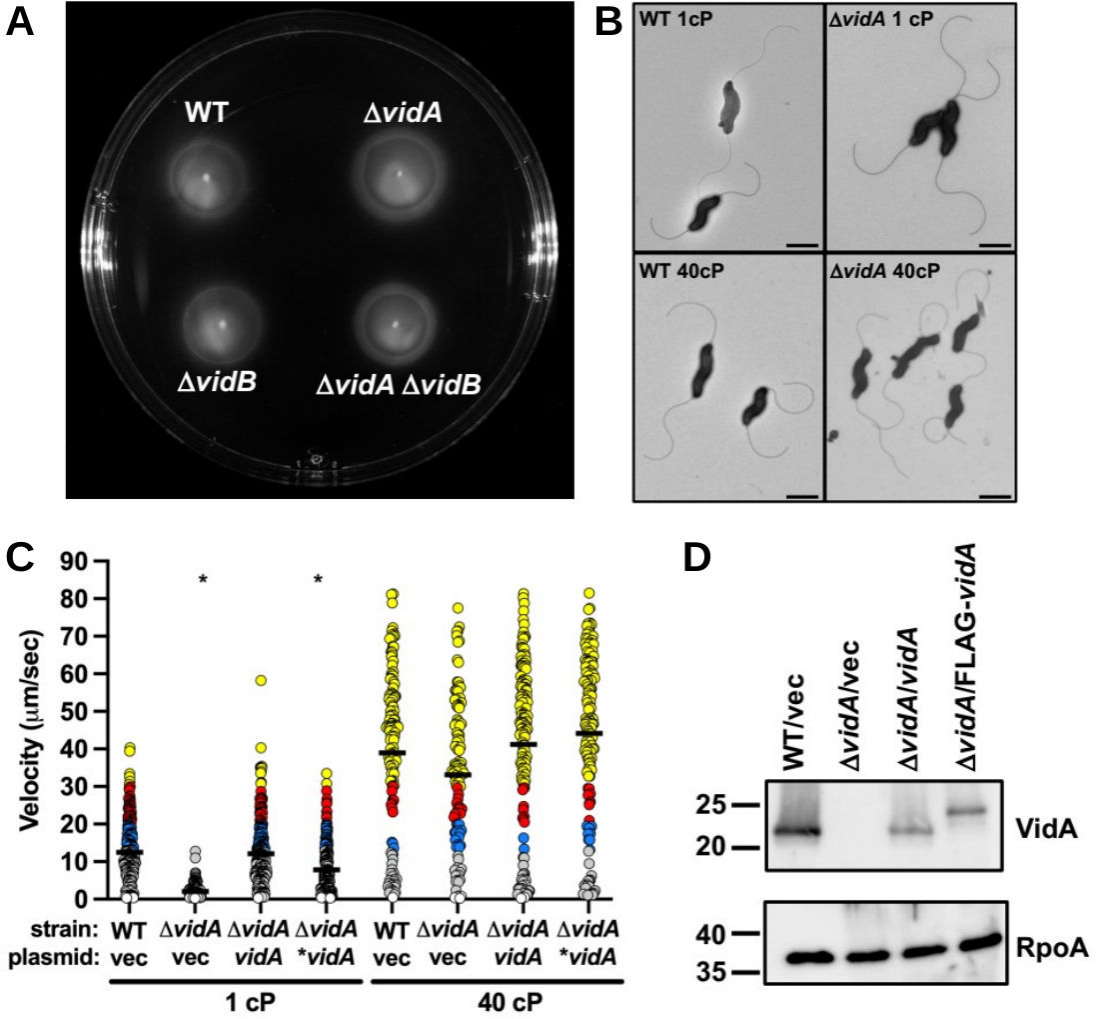
Flagellation and motility phenotypes of *C. jejuni* Δ*vidA*. (**A**) Motility assay in semi-solid motility agar. Motility assays of WT *C. jejuni* and isogenic Δ*vidA* and Δ*vidB* mutants were performed by stabbing cultures of similar optical densities into MH motility medium containing 0.4% agar. Plates were incubated in microaerobic conditions at 37°C for 30 h. (**B**) Electron micrographs of WT *C. jejuni* and *C. jejuni* Δ*vidA* grown in media of different viscosities. After overnight growth in MH broth alone (1 cP) or MH broth with methylcellulose (40 cP), strains were visualized by transmission electron microscopy. Bar = 1 µm. (**C**) Swimming velocities of *C. jejuni* Δ*vidA* upon in *trans* complementation with WT *vidA*. Swimming velocity of WT with vector (vec) alone, Δ*vidA* with vector alone, Δ*vidA* with vector encoding WT *vidA*, and Δ*vidA* with vector encoding an N-terminal FLAG-tagged VidA (*vidA) after growth for 24 h in MH broth alone (1 cP) or MH broth with methylcellulose to achieve a viscosity of 40 cP. Swimming velocities of individual cells (*n* > 100) were measured by video tracking under dark-field microscopy. Assays were performed in triplicate and combined. Circles represent individual cells with velocities of <1 µm/s (white), 1–13 μm/s (gray), 13–20 μm/s (blue), 20–30 μm/s (red), and >30 µm/s (yellow). The statistical significance of the difference in swimming velocities between the WT strain with the vector alone and other strains at both 1 and 40 cP was calculated using one-way ANOVA followed by Tukey’s multiple comparisons test. *P < 0.05. (**D**) Immunoblot analysis of VidA in whole-cell lysates of WT *C. jejuni* or *C. jejuni* Δ*vidA* containing vector (vec) alone or vectors encoding WT *vidA* or N-terminal FLAG-tagged VidA (*vidA). Specific antiserum to VidA was used. Detection of RpoA serves as a control to ensure equal loading of proteins across strains.

Previous studies identified Cjj81176_0996 (Cj0977 in *C. jejuni* NCTC11168, hereafter referred to as VidA (based on findings described below) as a protein required for WT levels of invasion of *C. jejuni* into intestinal epithelial cells and colonization of the chick intestinal tract (86–88). The respective gene is a member of the σ^28^-dependent regulon and co-transcribed during the last stage of flagellar assembly with genes encoding proteins that compose the flagellar filament, such as the major flagellin FlaA (86, 88, 89). A crystal structure for VidA revealed a homodimer of subunits with a hotdog fold and a potential binding site for an acyl-CoA compound (87). However, this structure did not reveal further insights into how VidA may contribute to invasion or colonization. Although mutants lacking *vidA* showed comparable levels of flagellar motility in semi-solid MH motility media [containing 0.4% agar (86, 88), Fig. 2A)], one report made a cursory observation that a *vidA* mutant was impaired for motility in liquid media (90). Therefore, we analyzed the swimming velocity of *C. jejuni* Δ*vidA* in media with differing viscosities. We found that Δ*vidA* was severely impaired for motility in MH broth alone (1 cP), with a mean swimming velocity of 2.1 µm/s (Fig. 1B; Movie S3). Only 1.6% of Δ*vidA* cells in the population demonstrated a velocity equal to or greater than the average for WT cells at 1 cP (15.9 µm/s, Fig. 1B and C). However, the mutant swam faster as viscosity increased with average swimming velocities of 5.3 µm/s at 2.5 cP, 21.7–26.6 μm/s at 5–20 cP, and 32.6 µm/s at 40 cP (Fig. 1B and C; Movie S4). Although no significant difference was observed in mean swimming velocities between WT and Δ*vidA* in media at 20 and 40 cP, the distributions of velocities of the populations were most similar only at 40 cP (Fig. 1A through C). Examination of flagellar structure by transmission electron microscopy did not detect any differences in flagellar number, length, or curvature between WT *C. jejuni* and Δ*vidA* grown in MH broth at 1 or 40 cP (Fig. 2B). In *trans* complementation of Δ*vidA* with a constitutive promoter to produce VidA or VidA with an N-terminal FLAG tag (FLAG-VidA) restored swimming velocity at 1 cP to WT levels and production of VidA (Fig. 2C and D). However, overexpression of VidA in WT *C. jejuni* from a plasmid did not increase swimming velocity in 1- or 40-cP media (data not shown). Our findings indicate that VidA is required for WT levels of swimming velocity in a low-viscosity, non-Newtonian fluid but is not required for WT swimming velocities in a high-viscosity, non-Newtonian fluid.

### Examination of swimming velocities of WT *C. jejuni* and *C. jejuni* Δ*vidA* with increasing viscosities in Newtonian fluids

Although other bacterial flagellates increase swimming velocity as viscosity in non-Newtonian fluids increases, swimming velocities of these bacteria decrease as viscosity in Newtonian fluids increases (44–47). Considering our observations above, we assessed whether WT *C. jejuni* increases its swimming velocity in a Newtonian fluid and whether *C. jejuni* Δ*vidA* would show similar differences in swimming velocities relative to WT in a Newtonian fluid at different viscosities. We attempted to grow *C. jejuni* in MH broth supplemented with viscosity agents to give media Newtonian properties, but we were unsuccessful, presumably due to an intoxicant present in the chemical preparation of the reagents. However, we could monitor by dark-field microscopy swimming velocities of *C. jejuni* strains first grown overnight in MH broth alone and then resuspended in fresh MH broth alone or MH broth with increasing concentrations of Ficoll, a viscosity agent that maintains the Newtonian behavior of MH broth across a range of viscosities.

In MH broth alone (1 cP), the mean swimming velocity of WT cells was 11.8 µm/s (Fig. 1D and F). The swimming velocity of WT *C. jejuni* almost doubled to 21.2–22.0 µm/s as viscosity increased to 2.5 and 5.0 cP and was still higher at 10 cP (17.0 µm/s, Fig. 1D and F). Unlike MH with methylcellulose, we observed that swimming velocity did decrease in MH with Ficoll at the highest viscosity tested (40 cP) and returned to that observed with MH broth alone. These findings reveal a significant difference from *E. coli*: WT *C. jejuni* increases its swimming velocity as viscosity increases, regardless if the fluid has Newtonian or non-Newtonian characteristics. In contrast, *E. coli* swimming velocity was observed to decrease even at 2.5 cP and continued to decrease as viscosity increases in a Newtonian fluid (44–47).

Similar to our observations of *C. jejuni* swimming velocity in the non-Newtonian MH broth containing methylcellulose, we found that the mean swimming velocity of *C. jejuni* Δ*vidA* increased as viscosity increased (Fig. 1E and F). Compared with a swimming velocity of 1.9 µm/s in MH broth alone, we observed swimming velocities of 5.4 µm/s in MH with Ficoll at 2.5 cP, 12.6 µm/s in 5-cP broth, and 18 µm/s in 10-cP broth (Fig. 1E and F). While the average swimming velocity of Δ*vidA* was lower than WT in media with Ficoll at 2.5 and 5.0 cP (Fig. 1D through F), swimming velocities of WT and Δ*vidA* were similar at 10 cP. Like WT *C. jejuni*, the average swimming velocity of Δ*vidA* decreased in MH with Ficoll at 40 cP (13.3 µm/s). These data indicate that like in non-Newtonian fluids, VidA is required for WT swimming velocities in low-viscosity media but is not required for motility in high-viscosity environments. Since we observed VidA to mediate these effects on swimming velocities at low viscosities in both Newtonian and non-Newtonian fluids, we conclude that VidA activity is sensitive to the viscosity of the environment rather than whether the fluid has Newtonian or non-Newtonian behavior. Due to the ease in growing *C. jejuni* in MH broth with methylcellulose and that this media with methylcellulose has non-Newtonian properties analogous to intestinal mucus of hosts where *C. jejuni* resides in nature, all remaining assays in this work were performed in MH broth with methylcellulose to increase viscosity of the media.

### Dynamics of alteration of swimming velocity upon a change in viscosity

**Fig 3:**
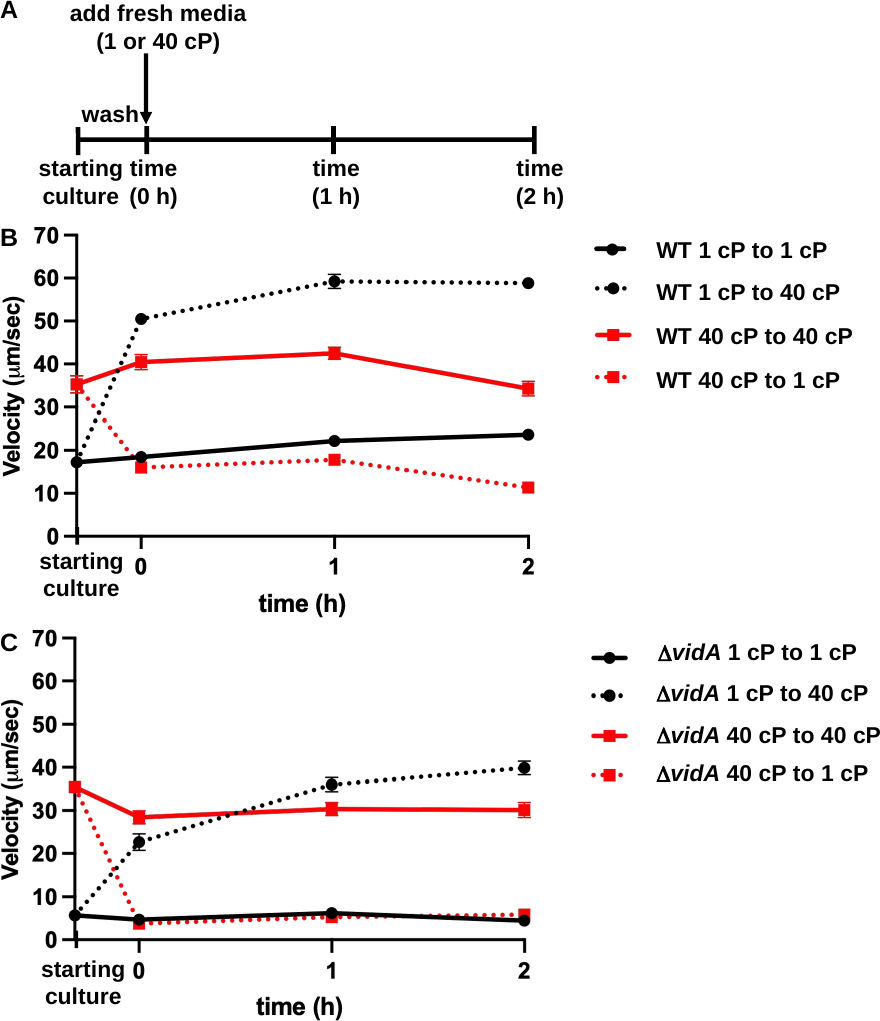
Changes in swimming velocity upon changes in extracellular viscosity over time. (**A**) Outline of experimental procedures. WT *C. jejuni* and *C. jejuni* Δ*vidA* were grown overnight in MH broth alone (1 cP) or with methylcellulose (40 cP) for starting cultures. Cultures were washed, split, and then resuspended in fresh MH media alone or with methylcellulose (40 cP) and incubated as standing cultures in microaerobic conditions at 37°C. Swimming velocities of individual cells (*n* > 100) in starting cultures or at times 0, 1, and 2 h after introduction into fresh MH media at 1 or 40 cP were measured by video tracking under dark-field microscopy. (**B and C**) Swimming velocities of WT *C. jejuni* (**B**) and *C. jejuni* Δ*vidA* (**C**) over time. For panels **B and C**, average swimming velocities (±standard errors) of cultures at each time are shown. Starting cultures represent cells after overnight growth in MH at 1 or 40 cP. Measurements at time 0 h are taken immediately after resuspension of washed cells in fresh MH media alone (1 cP) or MH with methylcellulose for 40 cP. Black solid lines with circles indicate cultures grown overnight in 1-cP media and introduced into fresh media of the same viscosity, whereas solid red lines with squares indicate cultures grown overnight into 40-cP media and introduced into fresh media of the same viscosity. Dotted black lines with squares indicate cultures grown overnight in 1-cP media and then switched to 40-cP media, whereas dotted red lines indicate cultures grown overnight in 40-cP media and then switched to 1-cP media.

With either WT *C. jejuni* or *C. jejuni* Δ*vidA*, we observed differences in swimming velocities after overnight growth in MH media at viscosities of 1 or 40 cP. We investigated whether viscosity-dependent differences in swimming velocities required growth and adaptation over time to the viscosity of the environment, or if changes in swimming velocity upon a change in viscosity occurred instantly. For this investigation, WT and Δ*vidA* were grown in MH broth alone at 1 cP or MH broth with methylcellulose to achieve 40 cP at 37°C as starting cultures. After overnight growth, cultures were washed, split equally, and then resuspended in fresh MH broth with a viscosity of 1 or 40 cP (Fig. 3A). Thus, bacteria were either introduced into fresh media with the same viscosity in which they were grown or introduced into fresh media at the alternative viscosity. Swimming velocities of standing cultures were then measured over time. As expected, WT and Δ*vidA* cells that were introduced into fresh media with the same viscosity in which they were grown retained the same swimming velocity over time (Fig. 3B and C). However, when WT or Δ*vidA* cells were introduced into media of the alternative viscosity, swimming velocities immediately changed. WT cells initially grown at 1 cP immediately swam with higher velocity in media at 40 cP (Fig. 3B). Similarly, WT cells initially grown in media at 40 cP immediately swam slower when given 1-cP media (Fig. 3B). We made similar observations with *C. jejuni* Δ*vidA* (Fig. 3C). Overall, these results suggest that modulation of swimming velocity in a viscosity-dependent manner involves mechanics that function on a relatively short time scale. Thus, these viscosity-dependent changes to swimming velocities likely do not require transcriptional changes or the production of new proteins to alter flagellar motor function.

### Suppressor analysis of *C. jejuni* Δ*vidA* for high-velocity swimming at low viscosity

**Fig 4:**
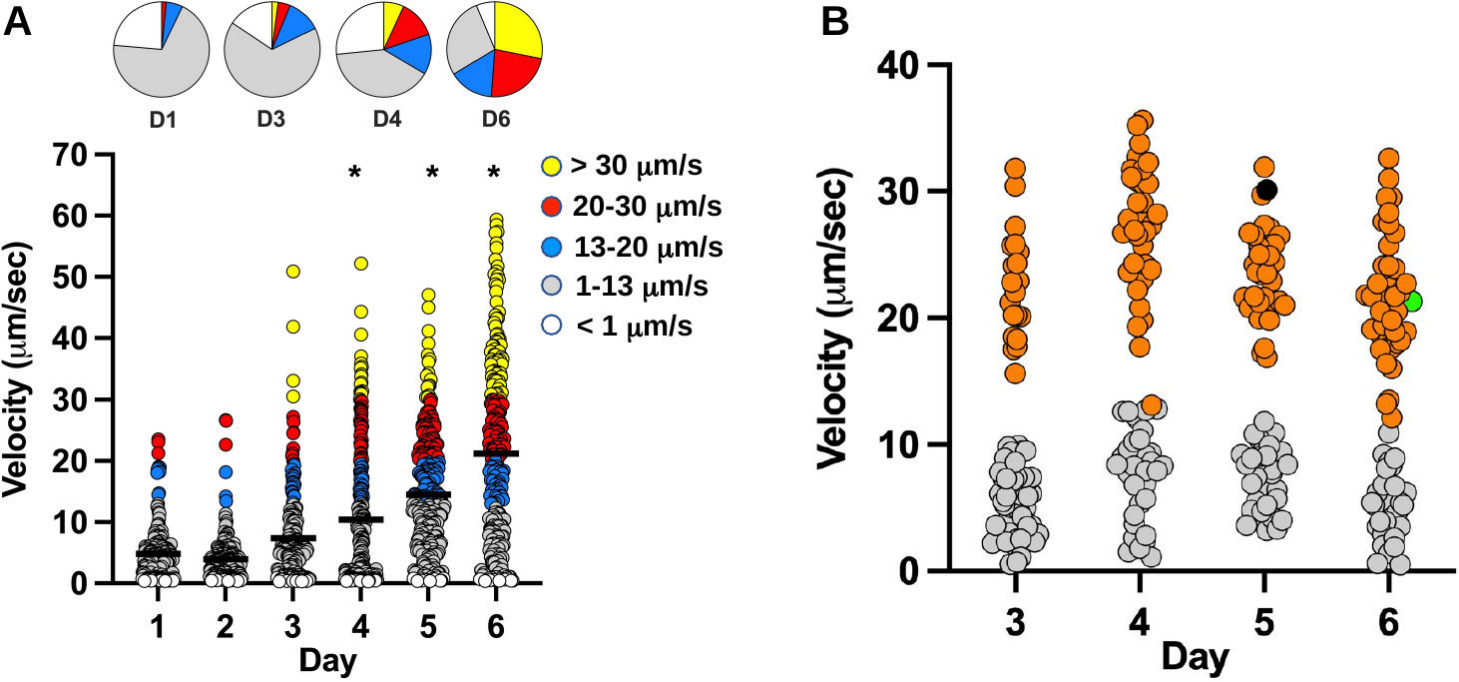
Identification of suppressor mutants of *C. jejuni* Δ*vidA* with increased swimming velocity upon passaging in low-viscosity media. (**A**) Swimming velocities of individual *C. jejuni* Δ*vidA* cells upon passaging in standing cultures of MH broth (1 cP) for up to 6 days. Swimming velocities of individual cells (*n* > 192) were measured by video tracking under dark-field microscopy each day. Bar represents the mean swimming velocity of the population. Circles represent individual cells with velocities of <1 µm/s (white), 1–13 μm/s (gray), 13–20 μm/s (blue), 20–30 μm/s (red), and >30 µm/s (yellow). Pie charts above represent portions of the population with different swimming velocities. Statistical significance of difference in swimming velocities of Δ*vidA* cells after day 1 vs cells from later cultures after passaging were calculated by one-way ANOVA followed by Tukey’s multiple comparisons test. *P < 0.05. (**B**) Swimming velocity of 72 *C*. *jejuni* Δ*vidA* isolates recovered at days 3–6 during passaging in standing cultures of MH broth (1 cP). Isolates with swimming velocities of >13 µm/s were analyzed by genomic sequencing or PCR to identify the mutation in the chromosome possibly responsible for the increased viscosity. Orange circles indicate individual cells with mutations in *vidB*. Gray circles represent isolates without *vidB* mutations, isolates that did not have increased viscosity in MH broth at 1 cP upon further analysis, or isolates that were not genetically analyzed. The black circle at day 5 represents one suppressor mutant with recombination in the *flaA* and *flaB* genes, but with WT *vidB*. The green circle at day 6 represents the suppressor mutant with the VidB_S363G_ point mutation.

To identify other factors that may modulate swimming velocity in low-viscosity environments, we pursued isolation of suppressor mutants of *C. jejuni* Δ*vidA* with higher swimming velocity in low-viscosity MH broth. For this analysis, we grew Δ*vidA* as standing cultures in MH broth alone (1 cP) for 24 h and passaged the cultures into fresh MH broth (1 cP) each day for up to 6 days. Swimming velocities of individual cells (*n* > 192) in the population were measured each day, and up to 72 random isolates were saved. During the first 2 days of passaging, the average swimming velocity of the population was between 4.0 and 4.8 μm/s (Fig. 4A). Between days 3 and 6, we observed a gradual increase in the mean swimming velocity of cells (10.4–21.2 µm, Fig. 4A). Suppressors with high swimming velocities above 30 µm/s also emerged during this period.

We measured the swimming velocities of 284 isolates recovered from days 3–6 of the procedure. We set a velocity of 13 µm/s in MH broth alone (1 cP) as a cut-off for isolates with potentially higher swimming velocities than the *C. jejuni* Δ*vidA* parental strain. At day 3, 27.8% of isolates showed velocities greater than 13 µm/s, and this percentage increased to 56.5% by day 6 (Fig. 4B). Overall, 44.7% of the isolates (127 total) demonstrated swimming velocities greater than 13 µm/s (Fig. 4B). Genomic sequencing of 15 of the high swimming velocity suppressor mutants revealed that 14 had alterations in a single gene, *Cjj81176_1107*, which shares fairly weak homology in a region to genes encoding diguanylate cyclases (DGCs) that generate cyclic diguanylate monophosphate (c-di-GMP) as a secondary messenger for signal transduction in many bacteria (91). The remaining Δ*vidA* suppressor mutant recombined parts of *flaA* and *flaB*, encoding the major and minor flagellins, respectively [Fig. 4B (92–94)]. This suppressor mutation was not analyzed further. Sequencing PCR products for *Cjj81176_1107* from the remaining 112 high swimming velocity suppressors revealed that all had mutations in *Cjj81176*_1107 (Table 1; Fig. 4B). In total, 99.2% of Δ*vidA* suppressor mutants with a higher swimming velocity in low-viscosity media than the parental Δ*vidA* strain had alterations in *Cjj81176_1107*. Due to our observations, we renamed *Cjj81176_1107* as *vidB* to be consistent in designating viscosity-dependent determinants of motility, analogous to our identification and reannotation of *vidA*.

**TABLE 1:** Identification of *C. jejuni* Δ*vidA* suppressor mutations

Δ*vidA* suppressor mutation in *Cjj81176_1107*	Position of mutation	Effect	No. of occurrences
Duplication of 17 nucleotides	20-bp 5′ to start	Promoter	1
T47C (L_16_S)^*a*^	T47	Nonsense^*b*^	1
G1158A (W_386_Stop)^*a*^	G158	Missense^*b*^	1
Deletion of 31 nucleotides	T196-A226	Frameshift	1
T_6_ tract to T_7_ tract	T231-T236	Frameshift	2
A_5_ tract to A_4_ tract	A331-A335	Frameshift	3
Insertion of 13 nucleotides	T610	Frameshift	1
G661T (E_221_Stop)^*a*^	G661	Missense^*b*^	1
A_7_ tract to A_8_ tract	A728-A734	Frameshift	1
A_7_ tract to A_6_ tract	A759-A766	Frameshift	23
A_7_ tract to A_8_ tract	A759-A766	Frameshift	4
A_4_ tract to A_3_ tract	A882-A885	Frameshift	1
AG_4_ tract to AG_3_ tract	A951-G958	Frameshift	64
AG_4_ tract to AG_5_ tract	A951-G958	Frameshift	16
T1087C (S_363_G)^*a*^	T1087	Nonsense	1
ATTT_1_ tract to ATTT_2_ tract	A1110-T1124	Frameshift	5
Duplication of 21 nucleotides	T1113	Frameshift	1

^*a*^ Effect of nonsense or missense mutation on protein sequence is indicated in parentheses.

^*b*^ Nonsense or missense mutation created an unstable protein in lysates as determined by immunoblot analysis.

**Fig 5:**
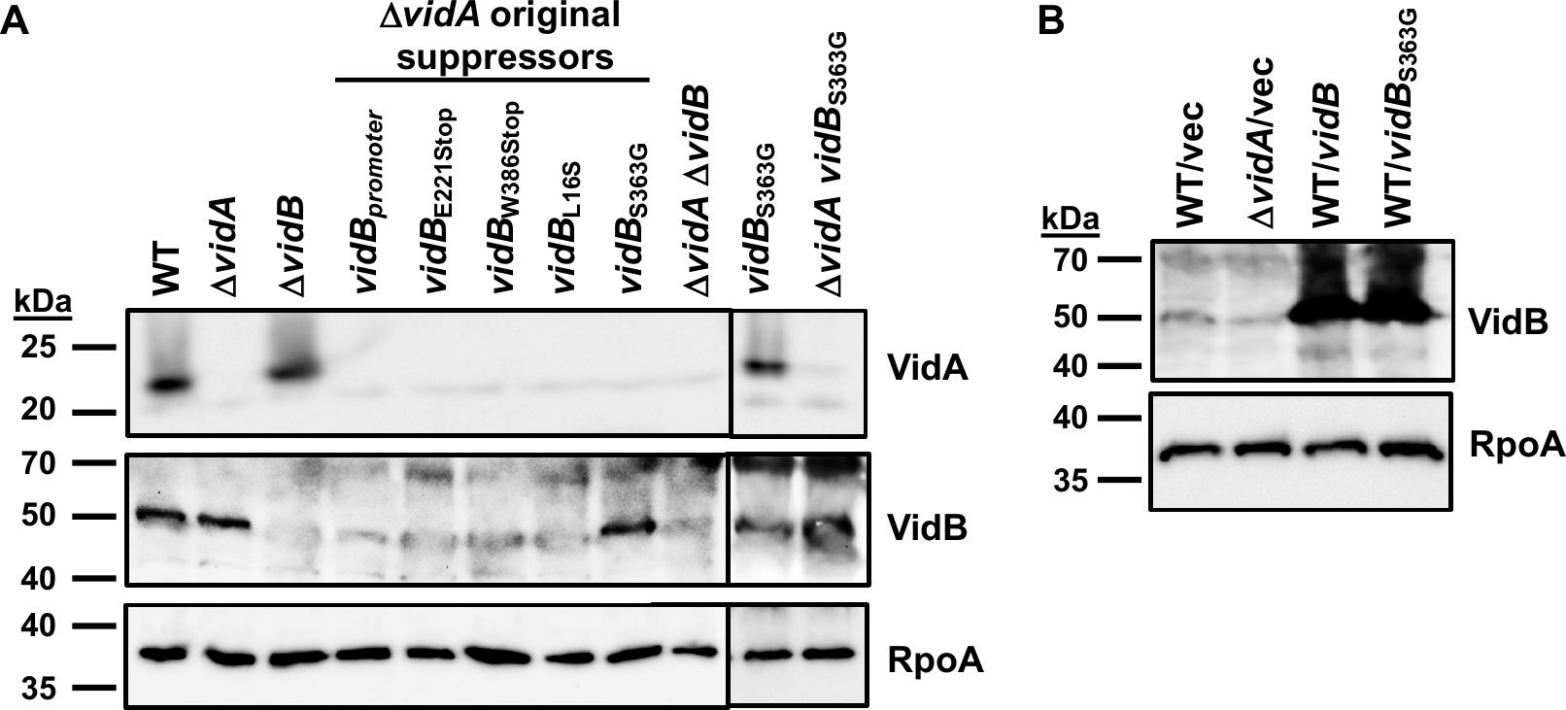
VidA and VidB levels in WT *C. jejuni* and mutants. (**A**) Immunoblot analysis of VidA and VidB in whole-cell lysates of WT *C. jejuni*, Δ*vidA*, Δ*vidA* suppressor mutants with higher swimming velocities, and *vidB* mutants constructed in WT *C. jejuni* or Δ*vidA*. (**B**) Immunoblot analysis of VidB in whole-cell lysates of WT *C. jejuni* or Δ*vidA* containing vector alone or vector to overexpress *vidB* or *vidB*_S363G_. For panels **A**and **B**, specific antiserum to VidA and VidB was used to detect each protein. Detection of RpoA serves as a control to ensure equal loading of proteins across strains. Note that a background band of ~50 kDa is observed around the same size of VidB in Δ*vidB* that hinders fully visualizing the absence of VidB in certain mutants.

Of the 127 suppressor mutations in *vidB*, 122 were frameshift mutations that altered the *vidB* coding sequence to result in a premature stop codon (Table 1). Another mutant had a mutation in the promoter of *vidB*, eliminating expression of the protein (Fig. 5A). Four other mutants were due to nonsense or missense mutations to result in VidB_E221Stop_, VidB_W386Stop_, VidB_L16S_, and VidB_S363G_. Analysis of whole-cell lysates (WCLs) from these mutants revealed that only the *vidB*_S363G_ mutant produced a VidB protein at similar levels as WT VidB (Fig. 4B and 5A). All other mutants failed to produce VidB mutant proteins either at the same level as WT or at the same stability as the WT VidB protein. We also observed that VidA and VidB were not required for the stability of each other as Δ*vidA* produced WT levels of VidB and Δ*vidB* produced WT levels of VidA (Fig. 5A).

### Validation of VidB for impacting swimming velocity at low viscosity

**Fig 6:**
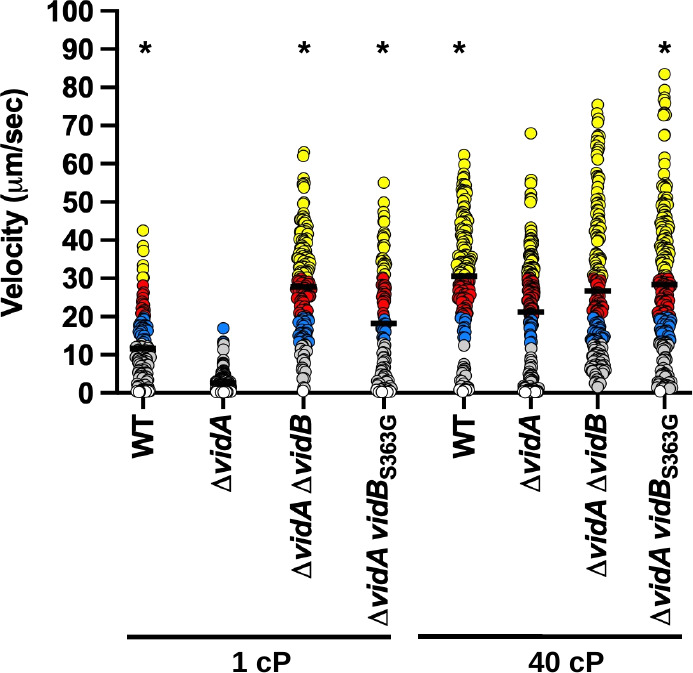
Swimming velocity of *C. jejuni* Δ*vidA* with or without *vidB* mutations at low and high viscosities. Swimming velocity of WT, Δ*vidA*, Δ*vidA* Δ*vidB*, and Δ*vidA vidB*_S363G_ after growth for 24 h in MH broth alone (1 cP) or MH broth methylcellulose (40 cP). Swimming velocities of individual cells (*n* > 100) were measured by video tracking under dark-field microscopy. Assays were performed in triplicate and combined. Bar represents the mean swimming velocity of the population. Circles represent individual cells with velocities of <1 µm/s (white), 1–13 μm/s (gray), 13–20 μm/s (blue), 20–30 μm/s (red), and >30 µm/s (yellow). Statistical significance of difference in swimming velocities of Δ*vidA* at 1 or 40 cP compared to other strains grown in media of the same viscosity was calculated by one-way ANOVA followed by Tukey’s multiple comparisons test. *P < 0.05.

We pursued a series of analyses to verify that VidB relieves the low swimming velocity of *C. jejuni* Δ*vidA*. Whereas the mean swimming velocity of the Δ*vidA* population was 2.6 µm/s in media at 1 cP, the Δ*vidA*Δ*vidB* mutant population swam with an average velocity of 27.8 µm/s (Fig. 6; Movie S5), verifying that mutations in *vidB* suppress the low-velocity defect of Δ*vidA* at low viscosity. Of note, Δ*vidA* Δ*vidB* had an average swimming velocity ~2.5-fold greater than WT *C. jejuni* at 1 cP (27.8 µm/s vs 11.6 µm/s, Fig. 6). The average swimming velocity of Δ*vidA*Δ*vidB* was similar and not statistically different in low and high viscosities (Fig. 6; Fig. S1; Movies S5 and S6). In addition, the mean swimming velocities of WT *C. jejuni* and Δ*vidA*Δ*vidB* were similar at 40 cP (Fig. 6), and we did not detect any differences in motility of the strains in semi-solid MH motility agar (Fig. 2A). Replacement of WT *vidB* with *vidB*_S363G_ in Δ*vidA* also relieved the swimming velocity defect of Δ*vidA* at 1 cP (Fig. 6). Multiple assays consistently showed that the Δ*vidA vidB*_S363G_ population tended to have an average swimming velocity modestly lower than that of Δ*vidA*Δ*vidB* (~18.2 vs 27.8 µm/s, Fig. 6; data not shown). The average swimming velocities of WT, Δ*vidA*Δ*vidB*, and Δ*vidA vidB*_S363G_ were equivalent to each other in media up to 40 cP (Fig. 6; Fig. S1). Although statistical significance varied with repeated assays, deletion or mutation of *vidB* in Δ*vidA* tended to cause higher swimming velocity in MH broth at 40 cP compared to the parental Δ*vidA* mutant.

**Fig 7:**
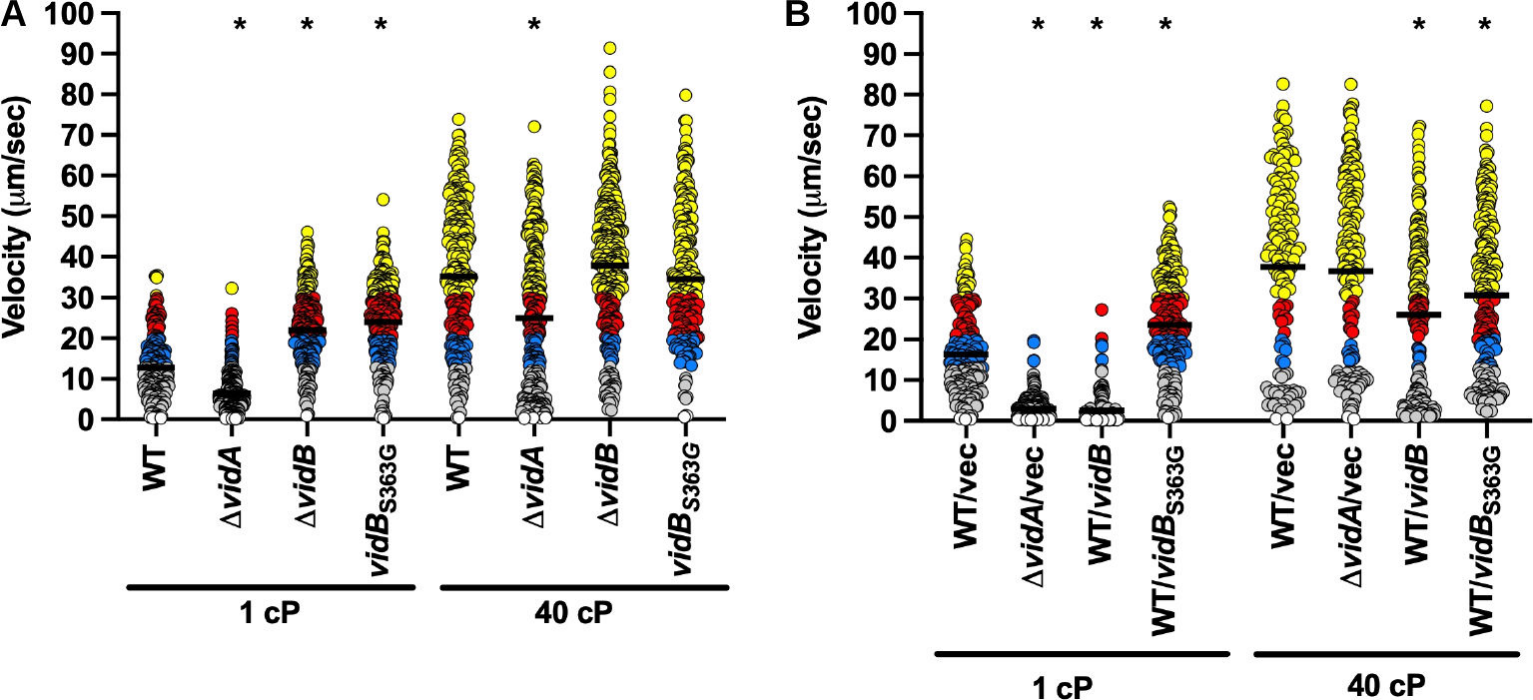
Swimming velocity of *C. jejuni* lacking *vidB* or overexpressing *vidB* at low and high viscosities. (**A and B**) Swimming velocity of cells after growth of cultures for 24 h in MH broth alone (1 cP) or MH broth with methylcellulose (40 cP). For panel **A**, strains included WT, Δ*vidA*, Δ*vidB*, and *vidB*_S363G_. For panel** B**, strains included WT with empty vector or vector containing *vidB* or *vidB*_S363G_ to overexpress the respective proteins and Δ*vidA* with the empty vector. Swimming velocities of individual cells (*n* > 100) were measured by video tracking under dark-field microscopy. Assays were performed in triplicate and combined. Bar represents the mean swimming velocity of the population. Circles represent individual cells with velocities of <1 µm/s (white), 1–13 μm/s (gray), 13–20 μm/s (blue), 20–30 μm/s (red), and >30 µm/s (yellow). Statistical significance of difference in swimming velocities of WT for panel** A** or WT with empty vector for panel **B **at 1 or 40 cP compared to other strains grown in media of the same viscosity was calculated by one-way ANOVA followed by Tukey’s multiple comparisons test. *P < 0.05.

Because mutation of VidB suppressed the swimming velocity defect of Δ*vidA*, we investigated whether VidB was sufficient to modulate flagellar motor output and swimming velocity in different viscosities for WT *C. jejuni*. The single Δ*vidB* or *vidB*_S363G_ mutant populations showed nearly a 100% increase in the average swimming velocity in media at 1 cP compared to the WT strain (Fig. 7A). However, the swimming velocities of WT and mutant strains in media at 40 cP were equivalent (34.6–37.9 µm/s; Fig. 7A). No apparent defect in motility was observed for Δ*vidB* relative to the WT strain in semi-solid motility agar (Fig. 2A).

We then determined whether overexpression of VidB could cause a reduction in swimming velocity for WT *C. jejuni*. For this approach, WT *vidB* or *vidB*_S363G_ was expressed from a strong promoter for the *C. jejuni* major flagellin (*flaA*) on a plasmid, in addition to expression of WT *vidB* from the native locus on the chromosome. Both the WT and mutant VidB protein were confirmed to be expressed at greater levels in WT *C. jejuni* than in WT *C. jejuni* with plasmid alone (Fig. 5B). Overexpression of WT *vidB* caused a severe reduction in swimming velocity when grown at 1 cP compared to the WT strain with plasmid alone (2.6 µm/s vs 16.2 µm/s, Fig. 7B; Movie S7). This low velocity due to *vidB* overexpression was comparable to that of Δ*vidA* with vector alone (Fig. 7B). In support of the S363G mutation in VidB isolated in the suppressor mutant analysis disabling activity of the protein, overexpression of VidB_S363G_ in WT *C. jejuni* failed to reduce swimming velocity at 1 cP, unlike WT VidB overexpressed in WT (Fig. 7B). Overexpression of WT VidB in WT *C. jejuni* was able to reduce the mean swimming velocity in media at 40 cP (37.8 µm/s vs 26.0 µm/s) but could not reduce the velocity as low as was observed with WT overexpressing VidB in media at 1 cP (26.0 µm/s vs 2.6 µm/s, Fig. 7B; Movie S8). Our data show that VidB alone, either by its removal in WT *C. jejuni* or Δ*vidA*, or by overexpression in WT *C. jejuni*, is sufficient to modulate swimming velocity, especially in low-viscosity conditions.

### Investigation of a linkage between VidB and c-di-GMP signaling for altering swimming velocity

VidB is predicted to be a cytoplasmic protein of 465 amino acids. Structural predictions and homology searches by PHYRE2 and other analyses predict a C-terminal domain (residues 289–444) most similar to the enzymatic domain of DGCs. In some bacterial flagellar systems, specific c-di-GMP binding proteins function as auxiliary brakes or clutches to alter flagellar motor function and swimming velocity under specific conditions (95–100). Clutch proteins of *Bacillus subtilis* prevent stators from contacting the rotor to decrease power and rotation (98, 100), whereas brake proteins in *E. coli* contact the rotor (or stators) to slow flagellar rotation (95, 96). However, most residues essential for DGC activity such as GTP and Mg^2+^ binding and catalysis are not conserved or very poorly conserved in VidB (101). Some c-di-GMP effector proteins have been postulated to be ancestral DGCs that have lost enzymatic activity but retained c-di-GMP binding at an inhibitory site [I site (102, 103)]. These proteins have neofunctionalized as c-di-GMP-dependent effectors to mediate specific biological functions. VidB also lacks a well conserved I site. Bioinformatic analysis did not detect any other regions of VidB with homology to domains with known functions.

Due to known flagellar brake or clutch proteins being influenced by c-di-GMP and VidB having activities that are consistent with a brake or clutch activity to modulate swimming velocity in a viscosity-dependent manner, we searched for any evidence of involvement in c-di-GMP in modulating VidB activity or swimming velocity in *C. jejuni. C. jejuni* is predicted to encode only one DGC, CbrR, with no obvious predicted partner phosphodiesterase to degrade c-di-GMP or effector protein evident in the genome. Previous work suggested that CbrR may have a negative influence on flagellar motility in *C. jejuni*, but it is unclear whether CbrR, which can bind GTP and c-di-GMP, is a bona fide DGC that synthesizes c-di-GMP in the *C. jejuni* cell (104).

We first analyzed CbrR, VidA, and VidB for *in vitro* c-di-GMP synthesis, c-di-GMP binding, or GTP binding activity. For this approach, we purified *Pseudomonas aeruginosa* WspR as a positive control for DGC activity and binding of the GTP substrate and c-di-GMP product (105). In *in vitro* differential radial capillary action of ligand assays (DRaCALAs), recombinant WspR and CbrR bound both GTP and c-di-GMP, but VidA and VidB did not (Fig. S2A). By thin-layer chromatography analysis of c-di-GMP generation from GTP, we could not observe DGC activity by CbrR or VidB, but we did observe c-di-GMP synthesis by the positive control WspR protein (Fig. S2B).

**Fig 8:**
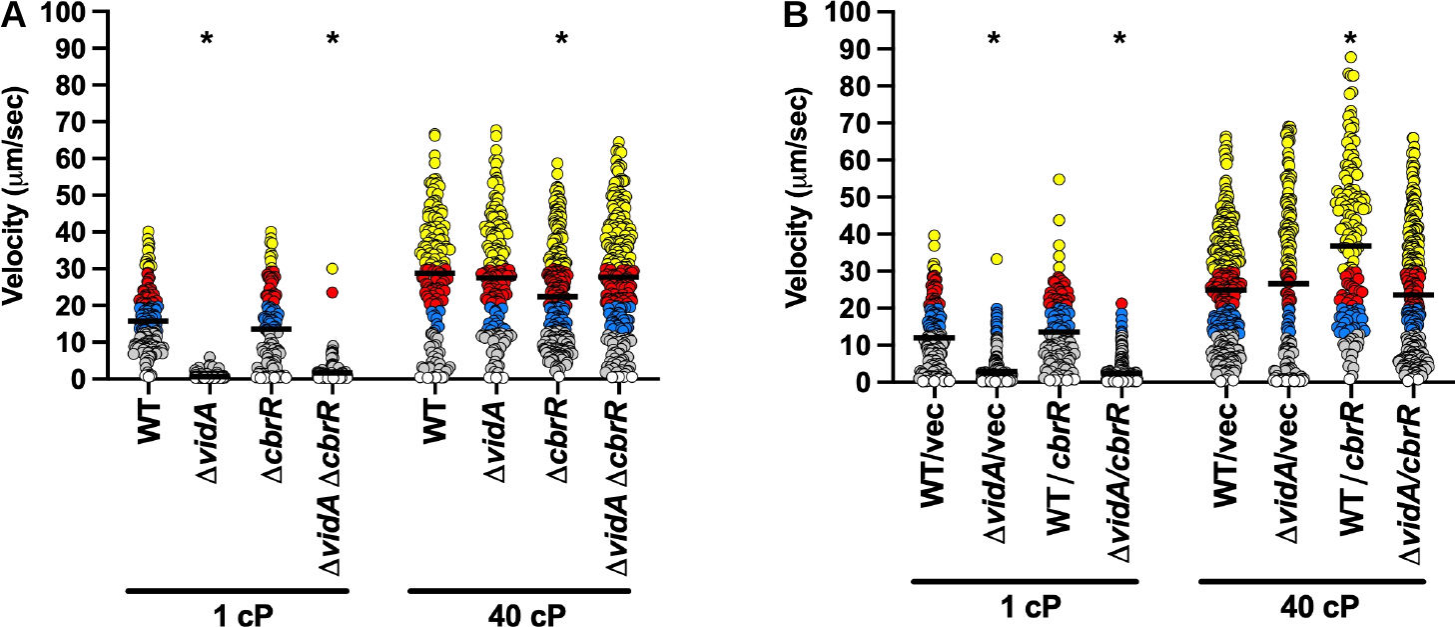
Swimming velocity of *C. jejuni* lacking *cbrR* or overexpressing *cbrR* at low and high viscosities. (**A and B**) Swimming velocity of cells after growth of cultures for 24 h in MH broth alone (1 cP) or MH broth with methylcellulose (40 cP). For panel** A**, strains included WT, Δ*vidA*, Δ*cbrR*, and Δ*vidA* Δ*cbrR*. For panel** B**, strains included WT or Δ*vidA* with the empty vector or vector containing *cbrR* for overexpression. Swimming velocities of individual cells (*n* > 100) were measured by video tracking under dark-field microscopy. Assays were performed in triplicate and combined. Bar represents the mean swimming velocity of the population. Circles represent individual cells with velocities of <1 µm/s (white), 1–13 μm/s (gray), 13–20 μm/s (blue), 20–30 μm/s (red), and >30 µm/s (yellow). Statistical significance in difference in swimming velocities of WT for panel** A** or WT with empty vector for panel** B** at 1 or 40 cP compared to other strains grown in media of the same viscosity was calculated by one-way ANOVA followed by Tukey’s multiple comparisons test. *P < 0.05.

We analyzed the effect of deleting *cbrR* (to decrease any possible c-di-GMP synthesis) or overexpressing CbrR (to increase any possible c-di-GMP synthesis) in both WT *C. jejuni* and Δ*vidA* on swimming velocity at low and high viscosities. Contrary to a previous report (104), we found that either deletion of *cbrR* or overexpression of *cbrR* slightly enhanced flagellar motility in semi-solid motility agar (Fig. S2C). The reason for this discrepancy with the previous report is unknown as the same strain of *C. jejuni* was used for both studies. Deletion of *cbrR* had no effect on swimming velocity of WT *C. jejuni* or Δ*vidA* in media at 1 cP or in Δ*vidA* at 40 cP (Fig. 8A). We did observe a slight decrease in swimming velocity upon deletion of *cbrR* in the WT strain (27.5 µm/s vs 22.4 µm/s) that was statistically significant; however, this difference was not consistently reproducible across replicate experiments (Fig. 8A). We did not observe any effect of overexpression of CbrR in WT *C. jejuni* or Δ*vidA* on swimming speeds at 1 cP (Fig. 8B). In media at 40 cP, we did observe an increase in swimming velocity when CbrR was expressed in WT *C. jejuni*, but this increase was not consistently observed across repeated experiments (Fig. 8B and data not shown). Together, these data suggest that unlike most previously discovered brake and clutch proteins that modulate the activity of the flagellar motor to alter motility in a c-di-GMP-dependent manner (95, 96, 98), VidB functions in a c-di-GMP-independent mechanism with VidA to control swimming velocity in a viscosity-dependent manner.

### Analysis of VidA and VidB for an impact on colonization

**Fig 9:**
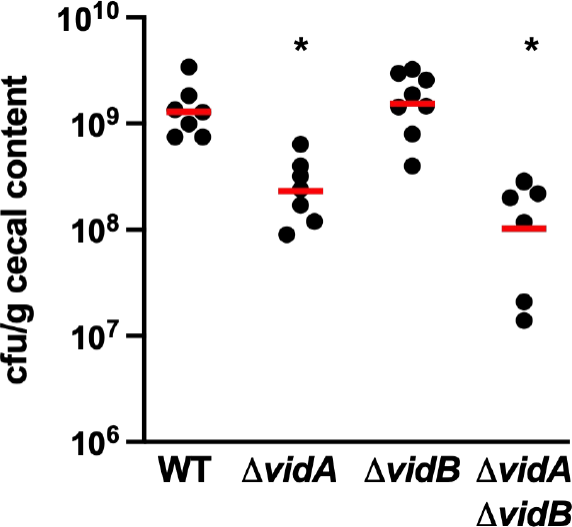
Colonization of WT *C. jejuni* and isogenic Δ*vidA* and Δ*vidB* mutants in the avian intestinal tract. Day of hatch chicks were orally infected with approximately 100 CFU of WT *C. jejuni*, and Δ*vidA*, Δ*vidB*, or Δ*vidA* Δ*vidB* mutants. Chicks were sacrificed at day 7 post-infection, and the levels of each *C. jejuni* strain in the ceca were determined (reported as CFU per gram of content). Each circle represents the level of *C. jejuni* in a single chick. Horizontal bars represent the geometric mean for each group. Statistical significance of difference in colonization capacity of strains to WT *C. jejuni* was determined using a two-tailed Mann-Whitney *U* test. *P < 0.05.

*C. jejuni* efficiently colonizes the mucus layer atop the intestinal epithelium of hosts, which includes the ceca and large intestines of avian species, which usually results in long-term colonization in the absence of disease, and the colon of humans to promote diarrheal disease (79, 81, 82). The bacterium encounters low-viscosity environments in the intestinal lumen and at the luminal surface of the intestines, but experiences higher viscosity as it swims deeper into the mucus layer toward the intestinal epithelial surface. We previously determined that VidA is required for WT levels of colonization of the chick ceca (88). Considering our analysis of the requirement of VidA for WT swimming velocity in low-viscosity conditions, it is unclear whether the colonization defect of Δ*vidA* is due to slower swimming speeds in low-viscosity environments that the bacterium likely encounters *in vivo*. In addition, we questioned whether the fairly unregulated high swimming velocity we observed *in vitro* in Δ*vidB* or Δ*vidA*Δ*vidB* may impact commensal colonization. Therefore, we inoculated day-of-hatch chicks with 100 CFU of WT and mutant strains and determined the level of *C. jejuni* in the chick ceca at day 7 post-infection. As we previously found (88), WT *C. jejuni* colonized the chick ceca at 1.30 × 10^9^-CFU/g cecal content, and Δ*vidA* showed a 5.6-fold colonization defect (~2.31 × 10^8^-CFU/g cecal content, Fig. 9). However, deletion of *vidB* from WT *C. jejuni* did not alter the colonization capacity. Likewise, the Δ*vidA*Δ*vidB* mutant only showed a colonization defect twofold lower than Δ*vidA*. While this colonization defect of Δ*vidA*Δ*vidB* was statistically significant compared to WT, it did not meet statistical significance when compared to Δ*vidA* (Fig. 8). These data suggest that the increased, relatively unregulated swimming velocity afforded by deleting *vidB* in *C. jejuni* does not impair the ability of the bacterium to efficiently colonize the chick ceca.

## DISCUSSION

As a resident of the lower intestines of many avian and other hosts and a pathogen invading the colonic epithelium of humans to promote diarrheal disease, *C. jejuni* is expected to have evolved strategies to colonize and maintain a presence in the viscous mucus layer atop the intestinal epithelium of hosts. It was first reported decades ago that the amphitrichous flagella of *C. jejuni* promote swimming with greater velocities in environments with higher viscosity than in those with lower viscosity (49–51). However, specific factors and mechanisms that modulate *C. jejuni* flagellar motor output to alter swimming velocity in a viscosity-dependent manner are largely unknown. In a recent investigation, we have reported that increased viscosity of the extracellular environments assists in proper wrapping of the amphitrichous flagella around the *C. jejuni* cell body to promote efficient motility and higher swimming velocity in higher viscosities compared to lower ones (85).

Other bacterial flagellates—both peritrichous organisms like *Salmonella* and *E. coli* and polar flagellates like *P. aeruginosa* and *Vibrio cholerae*—are known to swim more rapidly as viscosity increases in non-Newtonian fluids. The exact mechanisms and physical forces of flagella in relation to the viscous non-Newtonian fluid that mediates this increase in swimming velocity are not fully understood, but shear thinning is one factor (46). In contrast, *E. coli* and *Salmonella* swimming velocities plateau and steadily decrease as viscosity increases in Newtonian fluids (44, 45, 47). These observations indicate that the non-Newtonian nature of viscous fluids and perhaps not viscosity itself contributes to the ability of bacterial flagellates to swim faster in comparison to low-viscosity fluids.

By analyzing swimming velocities in both Newtonian and non-Newtonian fluids of increasing viscosity, we uncovered aspects of *C. jejuni* swimming motility that suggest the *C. jejuni* flagellar motor is mechanistically different from the model flagellar system of *E. coli* in altering motor output and swimming velocities in a viscosity-dependent manner. First, we found that *C. jejuni* swimming velocity increased in MH broth of increasing viscosity with either Newtonian or non-Newtonian properties. Thus, this increase in *C. jejuni* swimming velocity is due to viscosity itself rather than whether the fluid has Newtonian or non-Newtonian behavior. We suspect a combination of the high-torque generated by the *C. jejuni* flagellar motor due to an increased number of stators positioned at a wide distance from the flagellar motor, the amphitrichous positioning of the flagellar motors, the proper wrapping of the flagella around the *C. jejuni* cell body, and perhaps the rotation and wobble of the helical *C. jejuni* cell body all contribute to how the flagella increase swimming velocity in both types of fluids as viscosity increases. Indeed, deeper analyses of the spatial physics and interaction of the *C. jejuni* cell body and its rotating flagella with the polymers of the viscosity agents will be required to understand how the *C. jejuni* flagellar motor is mechanistically different from others in viscous environments to promote efficient swimming motility.

Second, we identified two factors—VidA and VidB—that appear to specifically modulate the swimming velocity of *C. jejuni* in a viscosity-dependent manner independent of whether the medium has Newtonian or non-Newtonian properties. VidA (Cjj81176_0996, Cj0977) was originally identified as a protein encoded within the *C. jejuni* σ^28^ regulon along with proteins that compose the flagellar filament and are produced at the last stage in flagellar biogenesis (86, 88, 89). However, VidA was not observed to be required for flagellation or flagellar motility in semi-solid motility agar (86, 88). Instead, VidA was found to be required for invasion of human intestinal epithelial cells and commensal colonization of the chick intestinal tract (86). An additional study observed that *C. jejuni* Δ*vidA* had a motility defect in liquid broth, which seemed to counter the original observations that VidA was not required for swimming in semi-solid motility agar (90). In this study, we more thoroughly analyzed the requirement of VidA for motility by using agents to increase the viscosity of liquid media and video-tracking analysis to measure velocity of swimming motility. By this approach, we established that VidA is required by *C. jejuni* for WT swimming velocity in low-viscosity media (1 cP, MH broth alone). Individual cells in the Δ*vidA* population rarely swam as fast at WT *C. jejuni* and the mean swimming velocity was five-fold lower than the WT population. The requirement for VidA for motility at low viscosity appears to be to repress or offset the function of VidB. All but one suppressor mutant in the *C. jejuni* Δ*vidA* background that swam with at least WT velocity in low-viscosity media had a mutation in *vidB*. At the lowest viscosity of media we analyzed (MH broth alone, 1 cP), WT *C. jejuni* swam faster than Δ*vidA*. Thus, without VidA counteracting the activity of VidB, VidB has a dominant, negative impact on motility of *C. jejuni* in low viscosity by reducing swimming velocity to very low levels. However, introducing either WT *C. jejuni* and Δ*vidA* to increasing viscosity in both Newtonian or non-Newtonian media resulted in augmented swimming velocities, although the mean swimming velocity of the Δ*vidA* population was lower than that of WT and Δ*vidB* in media of the highest viscosity. These findings suggest that viscosity alone does not fully counteract the negative effect of VidB activity on flagellar motor output and swimming velocity. Thus, two factors are required to offset the impact of VidB on the flagellar motor to reduce swimming velocity, the intrinsic VidA protein and the extrinsic viscosity of the environment.

One potential caveat in our analysis of enhanced swimming velocity in the presence of viscosity agents to create Newtonian and non-Newtonian fluids is the possible contamination of the polymer preparations with small carbon sources, which may be metabolized by some bacteria to enhance the proton-motive force and subsequent swimming velocity (46, 47). This was particularly important in previous studies in which dialysis removed impurities from polymers that contributed to enhanced swimming velocity of bacteria in motility buffers lacking nutrients (or very minimal nutrients) (47). We cannot fully discount the presence of impurities impacting the proton-motive force of *C. jejuni* when polymers were added to media to enhance swimming velocity as we used undialyzed polymers in our analyses. However, one consideration against this point is that the *C. jejuni* lacking VidB displayed high swimming velocity regardless whether any polymer was added to media, indicating that any such impurity, if present, did not further enhance the proton-motive force to increase swimming velocity. Furthermore, unlike previous analyses (47), our analysis was performed by adding polymers to MH media, which is a complex media that robustly supports *C. jejuni* growth, and any contaminating impurities in the polymer preparations likely do not significantly boost the proton-motive force to greatly enhance the swimming velocities we observed.

Removal of *vidB* from WT *C. jejuni* or Δ*vidA* resulted in unregulated flagellar motors mediating maximal swimming velocities in media with either low or high viscosities. This finding provided an intriguing revelation for the evolution of the *C. jejuni* flagellar motor. We propose that *C. jejuni* has evolved a flagellar motor that, by default, is naturally hardwired for increased power and generation of torque for a high velocity of swimming in viscous environments. Unlike flagellar motors of many other bacterial species that function optimally at low viscosity especially in Newtonian fluids, the normal activity of the *C. jejuni* flagellar motor allows for rapid propulsion of bacterial cells through viscous environments and actually requires evolution of proteins such as VidA and VidB to modulate flagellar motor output to slow down swimming velocity in low-viscosity environments (48, 49). The evolution of a naturally high-torque flagellar motor that needed to evolve auxiliary proteins for optimal motility in low viscosity likely reflects the natural habitat of *C. jejuni. C. jejuni* is found mainly in the thick mucus layer lining the lower intestinal tract of avian and other hosts. Its microaerobic growth requirements and an optimal growth temperature of 42°C further support the lower intestinal tracts of avian hosts, which have a body temperature of 42°C, as the primary natural reservoir for *C. jejuni*. Maintaining persistence in the viscous mucus layer atop the intestinal epithelium of avian and animal hosts for colonization would require a flagellar motor equipped to constantly propel the bacterium in this thick milieu and resist peristalsis for potential excretion by the intestines. Having a flagellar motor naturally hardwired to generate high torque for high swimming speeds in viscous media is consistent with our reported *in situ C. jejuni* flagellar motor structures revealed by cryo-electron tomography (52). Our previous work showed that *C. jejuni* produces one of the most complex bacterial flagellar motors with scaffolding structures and a rotor with a wider diameter that allows for an increased number of stator units per motor and their placement at a wider distance from the axle (52). The *C. jejuni* flagellar motor appears to maintain maximal stator occupancy with 17 stators incorporated per motor, unlike that of *E. coli* that has a dynamic number of stators from 1 to 11 that directly correlate with the amount of extracellular load on the flagellar filament (58, 59, 61). These alterations in stator number and placement in the *C. jejuni* flagellar motor presumably contribute to increased power and torque for flagellar rotation. Although *C. jejuni* can exist outside of hosts in feces, water sources, milk, or surface of foods for transmission from one host to another, these instances are presumably transient and likely at lower viscosities relative to the natural association with the host lower intestines. As such, VidA and VidB may be necessary to slow down the flagellar motor for low-viscosity conditions for relatively brief periods of time in the lifestyle of the organism outside of hosts. Thus, VidA and VidB are not essential for motility in the natural high-viscosity environments encountered by the bacterium. Instead, these proteins are auxiliary factors that modulate swimming velocity in alternative low-viscosity conditions.

Although we propose that VidA and VidB have evolved to slow swimming velocities in low-viscosity conditions, we have yet to observe a fitness disadvantage of a *vidB* mutant that naturally swims at an unregulated, maximal velocity in a low-viscosity environment. Efficient swimming in low viscosity conceivably requires fewer than the 17 stator units that appear to be consistently associated with the *C. jejuni* flagellar motor. Thus, fully powering flagellar rotation via proton transport by all 17 stator units in the *C. jejuni* motor could have a negative impact on the proton motive force and generation of the highest amount of cellular energy possible in low viscosity. For our assays, we grew *C. jejuni* in rich media with plentiful nutrients for energy, which may compensate for any drain on generating energy due to fueling the powerful flagellar motor of the *C. jejuni* Δ*vidB* mutant in low viscosity. It is possible that during transmission from one host to another, Δ*vidB* swimming at unregulated, maximal velocity may be less fit in a low-viscosity, nutrient-poor reservoir. Thus, we only speculate that VidA and VidB may have evolved to conserve energy in such environments.

While our study has provided original findings into viscosity-dependent modulations of swimming velocity in bacteria, we have been unable to reveal a mechanism by which VidB modulates flagellar motor output to impact swimming velocity and how VidA and external viscosity counteracts the activity of VidB. Since removal of VidB increases swimming velocity and overexpression of VidB in WT reduces swimming speeds, VidB activity is consistent with those of brake and clutch proteins found in flagellar motors of other bacterial species. Thus, VidB might interact with the rotor or stator components to separate connections of the stators and rotor [as a clutch (98–100)] or to exert force on the stators and/or rotor to stop rotation [as a brake (95–97)]. However, we have been unable to detect an interaction between VidB and the FliG rotor protein or the MotA and MotB stator proteins (data not shown). Furthermore, we have not been able to determine interactions of VidB with any other protein due to the inability to add an epitope tag to VidB (on either terminus or internally) for co-immunoprecipitation experiments that maintain VidB function to alter swimming velocity in a viscosity-dependent manner. We also attempted to overexpress VidB in the WT strain and isolate suppressor mutants that acquired the ability to swim at low viscosity, reasoning that we may find a mutation in a protein that is the target of VidB to lower flagellar motor output and swimming velocity. However, suppressor mutants that arose had acquired mutations in *vidB* itself, even though *vidB* was present at its normal chromosomal location and also overexpressed from a plasmid in *trans*. We have been able to co-immunoprecipitate proteins with a functional FLAG-VidA protein, but we did not acquire any evidence that VidA or VidB interact (data not shown). If VidA and VidB compete for docking to a motor component such as the rotor to alter swimming velocity, there may not be a direct interaction between VidA and VidB. Considering that both VidA and the extrinsic viscosity of the environment modulate the VidB activity, we may gain insight into how VidA, VidB, and viscosity impact the structure and components of the flagellar motor, specifically the stator and rotor parts, by analyzing the *in situ* structures of the *C. jejuni* flagellum in WT and mutant cells grown in low and high-viscosity media in subsequent exploration.

Since some bacterial flagellar brake and clutch proteins are c-di-GMP-dependent effector proteins (95, 96, 98), we investigated whether there was any role in c-di-GMP in modulating swimming velocity with VidB. This also allowed us to fully explore fairly weak sequence and structural predictions from bioinformatic analysis that VidB may have a C-terminus with an ancestral origin from bacterial DGCs that generate c-di-GMP. Curiously, the Δ*vidA* suppressor mutant that had a point mutation in VidB that created a stable protein is located near the poorly conserved GGDEF motif that is required to generate c-di-GMP by DGCs (101). However, we found no evidence that recombinant VidB could bind GTP or c-di-GMP as substrates or products of DGC activity. We also detected no *in vitro* DGC activity for VidB. Only one DGC, CbrR, is predicted to be encoded in the *C. jejuni* genome, but it is unclear if CbrR actually produces c-di-GMP in *C. jejuni* and may instead be a c-di-GMP binding effector (104). This previous report also suggested that CbrR might have an impact on flagellar motility, with deletion of *cbrR* causing increased flagellar motility and overexpression causing reduced flagellar motility in semi-solid motility agar. However, we were unable to replicate these findings as both our *cbrR* mutant and *cbrR* overexpressed strain caused modest increases in flagellar motility. There is always the possibility that another enzyme encoded in the *C. jejuni* genome could generate c-di-GMP or another secondary messenger that modulates VidB activity. However, we observed in this work instantaneous shifts from low to high velocity of swimming motility and vice versa upon a change in viscosity. Thus, there was no time delay for changes that would be expected with adaptation requiring the generation and accumulation of a second messenger to influence VidB activity. The simplest explanation is that modulation of VidB activity is independent of a second messenger and most likely relies on VidA and extrinsic viscosity. This postulation is consistent with the unique combination of VidA and VidB encoded in the genomes of *Campylobacter* species and not in others.

We analyzed whether the flagellar motors of Δ*vidB* cells that function at maximal swimming velocity in both low and high viscosity might alter the colonization capacity of *C. jejuni* for the natural avian hosts. However, compared to the WT strain, the Δ*vidB* mutant colonized the ceca of chicks at similar levels out to 7 days of infection. We previously observed that Δ*vidA* did have a commensal colonization defect for the chick ceca (88). Considering that our current study verified that the Δ*vidA* mutant swims slowly at low viscosity, we analyzed whether deletion of *vidB* in Δ*vidA* may rescue the colonization defect since Δ*vidA*Δ*vidB* swims with maximal swimming velocity regardless of viscosity. Curiously, the Δ*vidA*Δ*vidB* mutant continued to show a similar colonization defect as Δ*vidA*. This finding suggests that the low swimming velocity phenotype of Δ*vidA* is not the reason for the defect in commensal colonization. We think this is a reasonable interpretation as the viscosity in the chick ceca is likely high enough to relieve VidB activity and promote higher swimming velocities in Δ*vidA* as we observed in our *in vitro* studies presented here. These findings suggest that VidA has another role for *C. jejuni* in promoting optimal commensal colonization of the chick intestinal tract. A previous report has indicated that VidA interacts with another protein of *C. jejuni*, FlgV, whose biological role has not been fully elucidated (106). A crystal structure of VidA suggests a dimer with a binding pocket at the dimer interface for an acyl-CoA-linked metabolite for another function (87), which may be important for VidA activity in commensal colonization of chickens and/or invasion of human colonic epithelial cells.

To our knowledge, we have identified some of the first intrinsic factors that influence a bacterial flagellar motor to modulate swimming velocities specifically in a viscosity-dependent manner. Flagella have been known to participate in mechanosensing by modulating flagellar motor activity and gene expression upon contact with a surface (107–111). Our observations with *C. jejuni* reported herein may suggest a type of mechanosensing that occurs with viscosity changes in liquid milieus, but perhaps is more akin to rheosensing but without an applied flow rate (112, 113). Currently, there is very little evidence that *C. jejuni* has a swarming phenotype on solid surfaces like other motile bacteria. What is striking about the structurally complex *C. jejuni* flagellar motor is that, by default, it generates high torque for a high velocity of swimming in viscous environments that often impede motility by less complex flagellar motors exemplified by model peritrichous organisms such as *E. coli* or *Salmonella* species, especially in viscous milieus with Newtonian behavior. Instead of requiring auxiliary proteins to enhance function of the flagellar motor for higher swimming velocities, *C. jejuni* has apparently evolved proteins to dampen the normal high-torque output of the flagellar motor to swim with lower velocity in less viscous environments. Continued exploration of this complex, high-torque flagellar motor, along with those produced by other bacteria such as *Vibrio*, *Helicobacter*, and *Borrelia* species, will undoubtedly provide new insights into adaptation of the mechanics of bacterial flagellar motors to propel bacteria through environments.

## MATERIALS AND METHODS

### Bacterial strains and plasmids

*C. jejuni* 81–176 strains created and used in this study are described in Table 2. All plasmids constructed or involved in creation of strains in these studies are described in Table 3. Prior to each experiment, *C. jejuni* strains were routinely grown from freezer stocks on MH agar in microaerobic conditions (85% N_2_, 10% CO_2_, and 5% O_2_) at 37°C for 48 h and then restreaked on MH agar and grown for 16 h under identical conditions. As required, antibiotics were added to MH broth or agar at the following concentrations: 10-µg/mL trimethoprim, 15-µg/mL chloramphenicol, 50-µg/mL kanamycin, 30-µg/mL cefoperazone, or 0.5-, 1.0-, 2.0-, or 5.0-mg/mL streptomycin. All *C. jejuni* strains were stored at −80°C in an 85% MH broth and 15% glycerol solution. *Escherichia coli* DH5α and DH5α/pRK212.1 were grown on Luria-Bertani (LB) agar or in LB broth containing 100-µg/mL ampicillin, 15-µg/mL chloramphenicol, 50-µg/mL kanamycin, or 12.5-µg/mL tetracycline as appropriate. All *E. coli* strains were stored at −80°C in an 80% LB broth and 20% glycerol solution.

**TABLE 2:** Bacterial strains used in this study

Strain	Genotype	Source/reference
*E. coli* strains		
DH5α	*E. coli supE44* ∆*lacU169* (φ80*lacZ*ΔM15) *hsdR17 recA1 endA1 gyrA96 thi-1 relA1*	Invitrogen
DH5α/pRK212.1	DH5α with conjugation transfer element	(114)
Top10	F− *mcrA* ∆(*mrr-hsdRMS-mcrBC*) φ80*lacZ*ΔM15 ∆*lacX74 recA1 araD139* ∆(*ara-leu*)7697 *galE15 galK16 rpsL* (Str^r^) *endA1 nupG*	Invitrogen
XL1-Blue	F′::Tn*10 proA^+^B^+^lacI^q^*Δ(*lacZ)M15/recA1 endA1 gyrA96* (Nal^r^) *thi hsdR17* (r_K_^−^ m_K_^+^) *glnV44 relA1 lac*	New England Biolabs
*Campylobacter jejuni* strains		
DRH212	81–176 *rpsL*^Sm^	(115)
DRH837	81–176 *rpsL*^Sm^/pECO102	(116)
ABT501	81–176 *rpsL*^Sm^Δ*vidA*	(88)
DAR5362	81–176 *rpsL*^Sm^ Δ*vidA/*pECO102	This study
DAR5364	81–176 *rpsL*^Sm^Δ*vidA/*pDAR5476	This study
DAR5615	81–176 *rpsL*^Sm^Δ*vidA/*pDAR5357	This study
DAR6160	81–176 *rpsL*^Sm^ *cbrR*::*cat-rpsL*	This study
DAR6162	81–176 *rpsL*^Sm^Δ*vidA cbrR*::*cat-rpsL*	This study
DAR6236	81–176 *rpsL*^Sm^Δ*vidA vidB*::*cat-rpsL*	This study
DAR6232	81–176 *rpsL*^Sm^ *vidB*::*cat-rpsL*	This study
DAR6249	81–176 *rpsL*^Sm^Δ*cbrR*	This study
DAR6307	81–176 *rpsL*^Sm^Δ*vidA*Δ*cbrR*	This study
DAR6357	81–176 *rpsL*^Sm^Δ*vidA* Δ*vidB*	This study
DAR6668	81–176 *rpsL*^Sm^Δ*vidA vidB*_S363G_	This study
DAR6408	81–176 *rpsL*^Sm^Δ*vidB*	This study
DAR6672	81–176 *rpsL*^Sm^ *vidB*_S363G_	This study
DAR6673	81–176 *rpsL*^Sm^/pDAR1604	This study
DAR6675	81–176 *rpsL*^Sm^Δ*vidA/*pDAR1604	This study
DAR6727	81–176 *rpsL*^Sm^/pDAR6580	This study
DAR6812	81–176 *rpsL*^Sm^Δ*vidA*/pDAR6606	This study
DAR6851	81–176 *rpsL*^Sm^/pDAR6301	This study
DAR6853	81–176 *rpsL*^Sm^Δ*vidA*/DAR6301	This study

**TABLE 3:** Plasmids used in this study

Plasmid	Genotype	Source/reference
pUC19	Amp^R^, general cloning vector	New England BioLabs
pQE30	AmpR, expression vector for adding 6XHis-tag to N-terminus of proteins	Qiagen
pBAD/myc-HisA		Invitrogen
pRY108	Kan^R^, *E. coli-C. jejuni* shuttle vector	(117)
pRY109	Source of *cat* cassette	(117)
pDRH265	Source of *cat-rpsL* cassette	(115)
pECO102	Cat^R^, *E. coli-C. jejuni* shuttle vector containing *cat* promoter and stop codon for expression of genes for complementation	Wiesner
pVL791	Contains wspR	(114)
pDAR964	Cat^R^, *E. coli-C. jejuni* shuttle vector containing *cat* promoter with an in-frame N-terminal FLAG sequence for expression of genes for complementation	(118)
pDAR1425	pRY108 with 206 bases of DNA containing promoter for *flaA* and start codon cloned into the XbaI and BamHI sites	This study
pDAR1604	pRY108 with 206 bases of DNA containing promoter for *flaA* and sequence for FLAG tag fused in-frame to the start codon cloned into the XbaI and BamHI sites	(119)
pDAR5357	pDAR964 with codon 2 to stop codon of *vidA* cloned into the BamHI site of pDAR964 to create a N-terminal FLAG tag fusion protein	This study
pDAR5476	pRY112 with 76 bases of DNA containing promoter for *cat* and start codon with in-frame BamHI site, FLAG tag epitope sequence, and stop codon cloned into the XbaI and PstI sites for expression of C-terminal FLAG-tagged proteins for complementation	This study
pDAR5571	pDAR5476 with codon 2 to penultimate codon of *vidA* cloned in-frame in BamHI site	This study
pDAR6131	pUC19 with DNA fragment to create in-frame deletion of codons 47–347 of *vidB* with 0.8 kb of upstream and downstream sequences cloned in to the EcoRI site	This study
pDAR6134	pQE30 with codon 2 to stop codon of *vidB* cloned into the BamHI site	This study
pDAR6144	pUC19 with DNA fragment to create in-frame deletion of codon 2 to 375 of *cbrR* with 0.8 kb of upstream and downstream sequences cloned in to the EcoRI site	This study
pDAR6141	pUC19 with DNA fragment containing *cbrR* with 0.8 kb of upstream and downstream sequences cloned into the EcoRI site; contains G522A mutation to create an EcoRV site within *cbrR*	This study
pDAR6152	SmaI *cat-rpsL* cassette cloned into the EcoRV site of *cbrR* in pDAR6141	This study
pDAR6158	pUC19 with DNA fragment containing *vidB* with 0.8 kb of upstream and downstream sequences cloned into the EcoRI site, contains C890A mutation to create an EcoRV site within *vidB*	This study
pDAR6203	pBAD/myc-HisA with codon 2 to penultimate codon of *cbrR* cloned into the NcoI and HindIII sites	This study
pDAR6214	SmaI *cat-rpsL* cassette cloned into the EcoRV site of *vidB* in pDAR6158	This study
pDAR6301	pDAR1425 with *cbrR* from codon 2 to the stop codon cloned into the BamHI site	This study
pDAR6535	pBAD/myc-HisA with codon 2 to penultimate codon of *wspR* cloned into the NcoI and HindIII sites	This study
pDAR6574	pUC19 with DNA fragment containing *vidB*_S363G_ with 0.8 kb of upstream and downstream sequence cloned into the EcoRI site	This study
pDAR6580	pDAR1425 with *vidB* from codon 2 to the stop codon cloned into the BamHI site	This study
pDAR6606	pDAR1425 with *vidB*_S363G_ from codon 2 to the stop codon cloned into the BamHI siteco	This study

### Construction of *C. jejuni* mutants

*C. jejuni* mutants were constructed by electroporation of plasmid DNA or natural transformation of *in vitro* methylated plasmid DNA following previously described methods (115, 120). All plasmids were constructed by ligation of DNA fragments into plasmids by T4 DNA ligase or Gibson Assembly Mastermix (New England BioLabs).

For construction of *vidB* mutants, DNA fragments with approximately 800-bp upstream and downstream of the *vidB* coding sequence were amplified by PCR from *C. jejuni* 81–176 genomic DNA. Primers were designed to create a C890A point mutation inside the *vidB* coding sequence to create an EcoRV restriction site. The DNA fragments were cloned into EcoRI-digested pUC19 to create pDAR6158. A SmaI fragment containing a *cat-rpsL* cassette from pDRH265 was then inserted into the EcoRV site in *vidB* in pDAR6158 to result in pDAR6214 (115). pDAR6214 was then introduced into DRH212 or ABT501, and potential transformants that replaced WT *vidB* with *vidB::cat-rpsL* were recovered on MH agar with chloramphenicol (88, 115). Mutations were verified by colony PCR to result in DAR6232 (81–176 *rpsL*^Sm^ *vidB::cat-rpsL*) and DAR6236 (81–176 *rpsL*^Sm^ Δ*vidA vidB::cat-rpsL*). A plasmid harboring an in-frame deletion of *vidB* was created by designing primers to amplify approximately 800 bases upstream and downstream of *vidB* and portions of *vidB* coding sequence to delete codons 47–347. These DNA fragments were fused and assembled into EcoRI-digested pUC19 to create pDAR6131. For cloning of *vidB*_S363G_, primers were used to amplify the *vidB* sequence from ABT501 suppressors 6–10 with approximately 800 bp of upstream and downstream sequence and cloned into EcoRI-digested pUC19 to create pDAR6574. pDAR6131 and pDAR6574 were introduced into DAR6232 and DAR6236. Transformants were isolated on MH agar with streptomycin and then screened for sensitivity to chloramphenicol. Colony PCR and DNA sequencing verified mutants that had replaced *vidB::cat-rpsL* with the Δ*vidB* or *vidB*_S363G_ on the chromosome to result in DAR6408 (81–176 *rpsL*^Sm^ Δ*vidB*), DAR6357 (81–176 *rpsL*^Sm^ Δ*vidA*Δ*vidB*), DAR6672 (81–176 *rpsL*^Sm^ *vidB*_S363G_), and DAR6668 (81–176 *rpsL*^Sm^ Δ*vidA vidB*_S363G_).

For construction of *cbrR* mutants, DNA fragments with approximately 800 bp upstream and downstream of the *cbrR* coding sequence were amplified by PCR from *C. jejuni* 81–176 genomic DNA. Primers were designed to generate a G522A point mutation inside the *cbrR* coding sequence to create an EcoRV restriction site. The DNA fragments were cloned into EcoRI-digested pUC19 to create pDAR6141. A SmaI fragment containing a *cat-rpsL* cassette from pDRH265 was then inserted into the EcoRV site in *cbrR* in pDAR6141 to result in pDAR6152 (115). pDAR6152 was introduced into DRH212 or ABT501, and potential transformants that replaced WT *cbrR* with *cbrR::cat-rpsL* were recovered on MH agar with chloramphenicol (88, 115). Colony PCR verified correct construction of mutants to result in DAR6160 (81–176 *rpsL*^Sm^ *cbrR::cat-rpsL*) and DAR6162 (81–176 *rpsL*^Sm^ Δ*vidA cbrR::cat-rpsL*). A plasmid containing an in-frame deletion of *cbrR* was created by designing primers to amplify approximately 800 bases upstream and downstream of *cbrR* and portions of *cbrR* coding sequence to delete codons 2–375 to create pDAR6144. This plasmid was then introduced into DAR6160 and DAR6162. Transformants were isolated on MH agar with streptomycin and then screened for sensitivity to chloramphenicol. Colony PCR verified correct mutants that had replaced *cbrR::cat-rpsL* with Δ*cbrR* on the chromosome to result in DAR6249 (81–176 *rpsL*^Sm^ Δ*cbrR*) and DAR6307 (81–176 *rpsL*^Sm^ Δ*vidA*Δ*cbrR*).

### Construction of plasmids for complementation studies

Multiple plasmids were constructed to allow for expression of genes from promoters of different strengths to result in different levels of proteins with or without epitope tags for complementation of mutants. Primers were designed to amplify by PCR from the genome of *C. jejuni* 81–176 a 206-bp fragment containing the promoter for *flaA* (encoding the FlaA major flagellin) and its start codon, which was followed by an in-frame BamHI restriction site. This fragment was cloned into the XbaI and BamHI sties of pRY108 to result in pDAR1425 (117). A similar fragment was amplified by primers so that DNA for an in-frame FLAG tag epitope is encoded after the start codon of *flaA*, followed by an in-frame BamHI restriction site. This fragment was cloned into the XbaI and BamHI sites of pRY108 to create pDAR1604 (117).

Plasmids containing coding sequences of specific genes were constructed to complement various mutants. Primers were designed to amplify the coding sequence of *vidA* from codon 2 through the stop codon with both 5′ and 3′ in-frame BamHI sties from the *C. jejuni* 81–176 genome. The resultant DNA fragment was cloned into the BamHI-digested pECO102 to create pDAR5629 and BamHI-digested pDAR964 to result in pDAR5357, which encodes VidA with an N-terminal FLAG tag (121). Primers were also designed to amplify by PCR the *vidB* or *vidB*_S363G_ coding from codon 2 to the stop codon of WT *C. jejuni* 81–176 or ABT501 suppressors 6–10, respectively, with 5′ and 3′ in-frame BamHI restriction sites. These DNA fragments were then inserted into BamHI-digested pDAR1425 to create pDAR6580 (encoding WT VidB) and pDAR6606 (encoding VidB_S363G_). Similarly, primers were designed to amplify the *cbrR* coding sequence from codon 2 to the stop codon with 5′ and 3′ in-frame BamHI restriction sites from *C. jejuni* 81–176. This DNA fragment was then inserted into BamHI-digested pDAR1425 to create pDAR6301.

All plasmids were transformed into *E. coli* DH5α/pRK212.1, which served as the donor strain for conjugation into *C. jejuni* (114). Plasmids were then conjugated into *C. jejuni* strains as previously described (122).

### Measurement of swimming velocity of individual cells

Methylcellulose (4,000 cP, Fisher) was added at various concentrations to MH broth immediately after autoclaving and stirred overnight to achieve a range of viscosities. Final viscosities were determined using a Gilmont falling ball viscometer. For measurement of swimming velocity of individual cells in MH broth of different viscosities, *C. jejuni* strains were grown under standard conditions overnight for 16 h on MH agar with appropriate antibiotics. Strains were resuspended from plates in MH broth without methylcellulose and diluted to an OD_600_ 0.8. One milliliter of culture was added to 20 mL of MH broth alone with appropriate antibiotics (no methylcellulose, 1 cP) or containing methylcellulose to create viscosities up to 40 cP. Cultures were incubated as standing cultures for 24 h at 37°C in microaerobic conditions. After growth, strains were diluted to an OD_600_ between 0.1 and 0.4 in the same viscosity of MH that they were grown. Samples of cultures were applied to microscope slides precleaned with 100% ethanol and then covered with a cover slip. Motility tracks of individual cells were visualized using an Olympus BX-40 microscope adapted for dark-field microscopy with a UPlanFL ×100 objective and recorded with an Orca-Spark (Hamamatsu) camera and HCImage Live software. Captured motility tracks of individual cells were subsequently processed for calculation of swimming velocity using ImageJ and an in-house python script. Velocity measurements for at least 100 cells were examined for each strain for each experiment. Each experiment was performed three times.

For analysis of swimming velocities in MH broth with Ficoll to increase viscosity, a 36% Ficoll 400 (Cytiva) stock solution was prepared in MH broth and sterilized. This solution was diluted with MH broth to achieve a range of viscosities as determined by a Gilmont falling ball viscometer. *C. jejuni* strains were grown as described above in MH broth alone (1 cP) for 24 h at 37°C in microaerobic conditions. After growth, strains were diluted to an OD_600_ between 0.1 and 0.4. One-milliliter samples were pelleted for 3 min by centrifugation and resuspended in fresh MH broth alone or MH broth containing Ficoll at different concentrations to expose cells to different viscosities. Samples of cultures were then examined by dark-field microscopy as described above.

To assess the temporal scale for adjusting swimming velocities upon a change in viscosity, WT *C. jejuni* and Δ*vidA* mutant strains were prepared as above and then grown as standing cultures for 24 h at 37°C in microaerobic conditions in MH broth alone (1 cP) or MH broth with methylcellulose (40 cP) in triplicate. Individual cells in each culture were analyzed by dark-field microscopy for determination of mean swimming velocities of the populations. Each culture was then diluted with 10 volumes of phosphate-buffered saline (PBS), centrifuged for 10 min at 5,500 × *g*, and washed again with 200 mL of PBS. After resuspension in PBS, cultures were split in half and centrifuged again. One pellet was resuspended in 20 mL of MH broth at the original viscosity, whereas the other pellet was resuspended in 20 mL of MH broth at the alternative viscosity. Cultures were incubated in microaerobic conditions at 37°C as standing cultures and analyzed immediately (time 0) or at 1 and 2 h upon incubation by dark-field microscopy to determine mean swimming velocities of the populations. The experiment was performed three times.

### Assessment of flagellar motility in semi-solid motility agar

After overnight growth on MH agar with appropriate antibiotics at 37°C in microaerobic conditions, strains were resuspended in MH broth and diluted to OD_600_ 0.8. Strains were then stabbed into MH motility agar containing 0.4% agar and appropriate antibiotics when required. Agar plates were then incubated at 37°C in microaerobic conditions for 30 h.

### Transmission electron microscopy

WT *C. jejuni* and *C. jejuni* Δ*vidA* were grown under standard conditions overnight for 16 h on MH agar with appropriate antibiotics. Strains were resuspended from plates in MH broth without methylcellulose and diluted to an OD_600_ 0.8. One milliliter of culture was added to 20 mL of MH broth alone with appropriate antibiotics (no methylcellulose, 1 cP) or containing methylcellulose to create a viscosity of 40 cP. Cultures were incubated as standing cultures for 24 h at 37°C in microaerobic conditions. One milliliter samples of culture were pelleted for 3 min at 13,200 rpm in a microcentrifuge, resuspended in 2.5% glutaraldehyde in 0.1-M cacodylate and then incubated on ice for 1 h. Copper-coated formvar grids were negatively glow discharged, and bacterial samples were then applied to the grids. The samples were stained with 2% uranyl acetate and visualized with an FEI Technai G2 Spirit Bio TWIN transmission electron microscope.

### Isolation of *C. jejuni* Δ*vidA* suppressor mutants with increased swimming velocity in low-viscosity media

*C. jejuni* Δ*vidA* [ABT501 (88)] was grown under standard conditions overnight for 16 h on MH agar. Bacteria were resuspended from plates in MH broth and diluted to an OD_600_ of 0.8. One milliliter of culture was added to 20 mL of MH broth alone (without methylcellulose, 1 cP) to achieve an OD_600_ of ~0.04 and incubated as a standing culture in microaerobic conditions at 37°C. After 24 h, a sample of culture was analyzed by dark-field microscopy to determine the swimming velocity of individual cells (*n* > 192) and the mean swimming velocity of the population. Dilutions of the culture were plated on MH agar to isolate individual colonies, which were stored at −80°C. Twenty milliliters of fresh MH broth was inoculated with the overnight culture to an OD_600_ of ~0.04, and the culture was incubated as a standing culture in microaerobic conditions at 37°C for another 24 h. Passaging of cultures into fresh MH broth without methylcellulose and subsequent dark-field microscopy analysis of swimming velocities of the populations were continued for 6 days. At the conclusion of the experiment, up to 72 individual potential suppressor mutants for each day from days 1 to 6 were stored at −80°C. Each potential suppressor mutant was then grown as individual cultures in MH broth alone (no methylcellulose, 1 cP) and analyzed by dark-field microscopy to determine its swimming velocity in MH broth (1 cP) relative to the WT *C. jejuni* and the parental Δ*vidA* strain.

Genomic DNA from the parental *C. jejuni* Δ*vidA* strain (ABT501) and 15 suppressor mutants that were verified to have a higher swimming velocity in low-viscosity media relative to Δ*vidA* was prepared. Purified genomic DNA was then treated with RNase If (New England BioLabs) and cleaned using the Zymo Genomic DNA Clean and Concentrator kit. The genomic DNA from above was used to generate bar-coded Bioo NEXTflex DNA libraries at the Indiana University Center for Genomics and Bioinformatics. These libraries were cleaned and verified using an Agilent 2200 TapeStation before pooling and sequencing on the Illumina NextSeq platform. Paired-end reads were de-multiplexed subsequent to analysis. Reads obtained from the parent (ABT501) and suppressor mutant genomes were mapped to the *C. jejuni* 81–176 reference genome (GCA_000015525.1) using Geneious R10.2.6. Single-nucleotide polymorphisms (SNPs) with a minimum variant frequency of 20% were identified in regions of the parent and suppressor mutant genomes with at least 5× coverage. SNPs identified in suppressor mutant genomes were individually compared to those identified within the parent genome. SNPs that were unique to the suppressor mutant genomes were presumed to be associated with the suppressor mutant phenotypes and investigated further. Identification of other suppressor mutants for which genomic sequencing was not applied involved PCR amplification of one or more suspected genes and then sequencing of PCR products.

### Purification of recombinant proteins

The coding sequence from codon 2 to the stop codon of VidB was amplified by PCR from *C. jejuni* 81–176 and then cloned by Gibson Assembly into BamHI-digested pQE30 to create pDAR6134. This plasmid was then moved to *E. coli* XL1-Blue for recombinant protein purification. XL1-Blue containing pDAR6134 was grown to mid-log phase in LB broth with shaking at 37°C and then induced with 1-mM isopropyl-beta-D1-thiogalactopyranoside (IPTG) for 3 h at 37°C.

*C. jejuni* 81–176 *cbrR* from codon 2 to the stop codon was amplified by PCR and inserted into NcoI and HindIII-digested pBadMyc/HisA by Gibson Assembly to create pDAR6203. *P. aeruginosa* WspR was amplified from pVL791 and cloned similarly in pBADMyc/HisA to create pDAR6535 (123). The plasmids were moved into *E. coli* Top10 for induction and purification. After growth to mid-log phase in LB broth, arabinose was added to a final concentration of 0.2% to induce expression of CbrR-Myc-6XHis and WspR-Myc-6XHis for 3 h at 37°C. 6XHis-VidA was induced from pABT522 as previously described (88).

After induction of proteins, cell pellets were collected by centrifugation and washed once with lysis buffer (50-mM NaH_2_PO_4_, 300-mM NaCl, 10-mM imidazole, pH 8.0). Cells were passed through an EmulsiFlex-C5 disruptor at 15,000–20,000 lb/in^2^ to lyse cells. The soluble fraction was recovered by centrifugation at 20,000 × *g* for 2 h. Recombinant 6XHis-VidB, CbrR-Myc-6XHis, WspR-Myc-6xHis, and 6XHis-VidA were purified via fast protein liquid chromatography (FPLC) using an NGC Chromatography System and Profinity IMAC columns (Bio-Rad) according to manufacturer’s instruction. Glycerol was added to a final concentration of 20% prior to storage at −80°C.

### Polyclonal antisera generation

Purified recombinant 6XHis-VidB was used to immunize guinea pigs by standard procedures for antisera generation via a commercial vendor (Cocalico Biologicals). All uses of animals in experimentation were approved by The Institutional Animal Care and Use Committee (IACUC) at the University of Texas Southwestern Medical Center.

### Immunoblotting analyses

Preparation of *C. jejuni* strains for WCLs was performed as previously described (116). All immunoblotting analyses of WCLs or immunoprecipitated proteins were performed with equal amounts of samples from *C. jejuni* strains after SDS-PAGE by standard procedures. For specific detection of proteins in WCLs, 10 µL of WCLs was analyzed to detect VidA and RpoA, and 20 µL of WCLs was analyzed to detect VidB. Proteins were detected with specific murine or guinea pig antisera generated previously or as described above (88, 124). Primary antisera were used at the following dilutions: VidA M155, 1:500; VidB UTGP296, 1:500; and RpoA M252, 1:2,000. All primary antisera were applied to immunoblots for 2 h. Appropriate horseradish peroxidase-conjugated goat antibodies were used as secondary antibodies to develop immunoblots.

### Analysis of c-di-GMP synthesis

Recombinant WspR, CbrR, and VidB were assessed for their ability to synthesize c-di-GMP from GTP following standard protocols (125). Briefly, MgCl_2_ was added to a final concentration of 10 mM to 40 µg of recombinant proteins. Reactions were started by addition of 10 µCi [α^32^P]-GTP, incubated at room temperature for 1 h, and then stopped with the addition EDTA to a final concentration of 250 mM. Thin-layer chromatography (TLC, BakerFlex Cellulose) plates were allowed to equilibrize to room temperature, and then 2 µL of the reactions was spotted onto the TLC plate. After spots had dried, the TLC plate was placed inside a sealed beaker containing approximately 250 mL of solvent (2:3 mixture of saturated NH_4_SO_4_ and KH_2_PO_4_). Once solvent migration had neared the end of the TLC plate, the plate was removed, dried, covered with a clear wrapping, and exposed to a phosphorimager cassette for 2 h or overnight.

### GTP and c-di-GMP binding analyses

Recombinant WspR, CbrR, VidA, and VidB were assessed for their ability to bind GTP and c-di-GMP by the DRaCALA (126). Briefly, 10× binding buffer (100-mM Tris-HCl, pH 8, 1-M NaCl, and 50-mM MgCl_2_), 10 µCi of [α^32^P]-GTP, and 14 µg of protein were added to a final reaction volume of 20 µL. Two microliters of each reaction was spotted onto nitrocellulose membranes and exposed to a phosphorimager for 30 min. For analysis of c-di-GMP binding, [^32^P]-labeled c-di-GMP was generated by recombinant WspR as described above and then heated at 95°C for 5 min to inactivate WspR. The reaction was then centrifuged at 20,000 × *g* for 5 min to separate WspR from [^32^P]-labeled c-di-GMP. DRaCALA reactions were performed as above except substituting [^32^P]-labeled c-di-GMP and spotting 6 µL of reaction mixture onto nitrocellulose membranes.

### Chick colonization assays

All uses of animals in experimentation were approved by IACUC at the University of Texas Southwestern Medical Center. The ability of wild-type *C. jejuni* or mutant strains to colonize the ceca of chicks after oral inoculation was determined as previously described (79). Briefly, fertilized chicken eggs (SPAFAS) were incubated for 21 d at 37.8°C with appropriate humidity and rotation in a Sportsman II model 1502 incubator (Georgia Quail Farms Manufacturing Company). Approximately 12–36 h after hatching, chicks were orally infected with 100 µL of PBS containing approximately 100 CFU of WT *C. jejuni* or an isogenic mutant strain. Strains were prepared for oral gavage by resuspension from MH agar after growth and dilution in PBS to an OD_600_ of 0.4 followed by serial dilution to obtain the appropriate inoculum for oral gavage of chicks. The number of CFUs of the inoculum was determined by serial dilution on MH agar. At day 7 post-infection, chicks were sacrificed and the cecal contents were recovered, weighed, and suspended in PBS to 0.1-g cecal content/mL PBS. Serial dilutions were spread on MH agar containing trimethoprim (TMP) and cefoperazone. Bacteria were grown for 72 h at 37°C in microaerobic conditions and then counted to determine the CFU per gram of cecal content for each chick. Statistical analyses were performed by the Mann-Whitney *U* test, with statistically significant differences between wild-type and mutant strains indicated with *P* values of <0.05.

## Data Availability

All data and methodologies are available upon request. Genomic sequences are available at the National Center for Biotechnology Information as part of Bioproject PRJNA987628, with individual genomic sequences assigned the following accession numbers: ABT501 (SRR25146826), ABT501 S1 (SRR25146825), ABT501 S2 (SRR25146820), ABT501 S3 (SRR25146819), ABT501 S4 (SRR25146818), ABT501 S5 (SRR25146817), ABT501 S6 (SRR25146816), ABT501 S7 (SRR25146815), ABT501 S8 (SRR25146814), ABT501 S9 (SRR25146813), ABT501 S10 (SRR25146824), ABT501 S11 (SRR25146823), ABT501 S12 (SRR25146822), and ABT501 S13 (SRR25146821).

## SUPPLEMENTAL MATERIAL

Supplemental materials 1 mbio.02544-23-s0001.docx Figures S1 and S2 and Movie S1-S8 legends.

Movie S1 mbio.02544-23-s0002.mpg WT swimming motility at low viscosity (1 cP).

Movie S2 mbio.02544-23-s0003.mp4 WT swimming motility at high viscosity (40 cP).

Movie S3 mbio.02544-23-s0004.mp4 *vidA* mutant swimming motility at low viscosity (1 cP)

Movie S4 mbio.02544-23-s0005.mp4 *vidA* mutant swimming motility at high viscosity (40 cP).

Movie S5 mbio.02544-23-s0006.mp4 *vidA vidB* mutant swimming motility at low viscosity.

Movie S6 mbio.02544-23-s0007.mp4 *vidA vidB* swimming motility at high viscosity (40 cP).

Movie S7 mbio.02544-23-s0008.mpg WT *C.jejuni* overexpressing VidB swimming motility at low viscosity (1 cP).

Movie S8 mbio.02544-23-s0009.mpg WT *C. jejuni* overexpressing VidB swimming motility at high viscosity (40 cP).

ASM does not own the copyrights to Supplemental Material that may be linked to, or accessed through, an article. The authors have granted ASM a non-exclusive, world-wide license to publish the Supplemental Material files. Please contact the corresponding author directly for reuse.
