# Supplementary material for: Viscosity-dependent determinants of Campylobacter jejuni impacting the velocity of flagellar motility
Source: mBio. 2023 Dec 12;15(1):e02544-23. doi: 10.1128/mbio.02544-23 (PMC10790790; doi:10.1128/mbio.02544-23)
Supplement: Supplemental materials 1 — Figures S1 and S2 and Movie S1-S8 legends. [file mbio.02544-23-s0001.docx]

**Supplementary Materials**

**Figure S1. Analysis of swimming velocities of WT and Δ*vidA* mutant strains with *vidB* mutations in media with different viscosities.** Swimming velocity of WT (gray), Δ*vidA* (white), Δ*vidA* Δ*vidB* (yellow), and Δ*vidA* *vidB*_S363G_ (blue) after growth for 24 h in (A) MH broth alone (1 cP) or MH broth with methylcellulose to achieve viscosities of (B) 2.5 cP, (C) 5 cP, (D) 10 cP, (E) 20 cP, and (F) 40 cP. Swimming velocities of individual cells (n > 100) were measured by video tracking under dark-field microscopy. Assays were performed in triplicate and combined. Data are presented as violin plots. The red bar represents the median and black bars represent the 25^th^ and 75^th^ quartiles. Statistical significance in swimming velocity of Δ*vidA* cells in cultures grown at each viscosity compared to other strains grown in media of the same viscosity was calculated by one-way ANOVA followed by Tukey’s multiple comparison test (* *p* < 0.05).


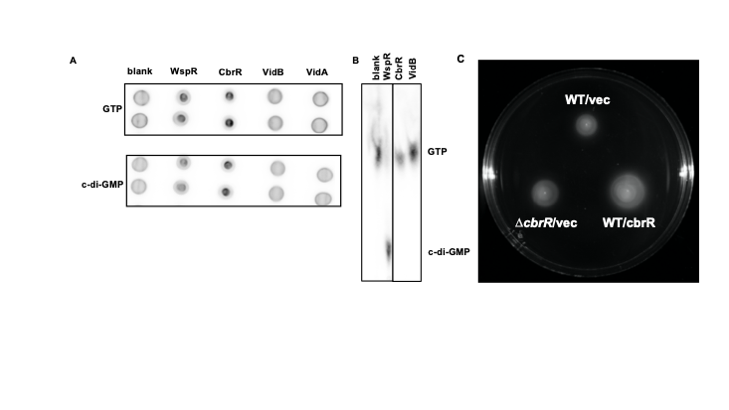


**Figure S2. Biochemical analysis of *C. jejuni* CbrR and its impact on motility.** (A) VidA, and VidB in purified proteins for GTP and c-di-GMP binding and c-di-GMP synthesis. (A) DRaCALA assay to analyze purified CbrR, VidA, and VidB proteins for GTP and c-di-GMP binding. Proteins were purified with Myc-6XHis-tags or 6XHis-tags and mixed with [α-^32^P]-GTP or ^32^P-c-di-GMP generated by WspR. Proteins were then immobilized on nitrocellulose membranes, washed, and exposed to a Phosphorimager for detection of GTP or c-di-GMP binding. Assays were performed in duplicate. (B) Thin-layer chromatography for c-di-GMP generation upon addition of [α-^32^P]-GTP to purified proteins. [α-^32^P]-GTP and generated ^32^P-c-di-GMP are indicated. For (A and B), *P. aeruginosa* WspR is used as a positive control. (C) Flagellar motility phenotypes of WT *C. jejuni* upon deletion or overexpression of *cbrR*. Motility assays were performed by stabbing cultures of WT *C. jejuni* containing vector (vec) alone, an isogenic Δ*cbrR* mutant with vector alone, or WT *C. jejuni* with a plasmid to overexpress *cbrR* at similar optical densities into MH motility medium containing 0.4% agar. Plates were incubated in microaerobic conditions at 37°C for 30 hours.

**Supplemental Movies**

**Movie S1. Flagellar motility of WT *C. jejuni* in low-viscosity media.** Dark-field microscopy of WT *C. jejuni* swimming in MH broth alone (1 cP).

**Movie S2. Flagellar motility of WT *C. jejuni* in high-viscosity media.** Dark-field microscopy of WT *C. jejuni* swimming in MH broth with methylcellulose (40 cP).

**Movie S3. Flagellar motility of *C. jejuni* Δ*vidA* in low-viscosity media.** Dark-field microscopy of *C. jejuni* Δ*vidA* swimming in MH broth alone (1 cP).

**Movie S4. Flagellar motility of *C. jejuni* Δ*vidA* in high-viscosity media.** Dark-field microscopy of *C. jejuni* Δ*vidA* swimming in MH broth with methylcellulose (40 cP).

**Movie S5. Flagellar motility of *C. jejuni* Δ*vidA* Δ*vidB* in low-viscosity media.** Dark-field microscopy of *C. jejuni* Δ*vidA* Δ*vidB* swimming in MH broth alone (1 cP).

**Movie S6. Flagellar motility of *C. jejuni* Δ*vidA* Δ*vidB* in high-viscosity media.** Dark-field microscopy of *C. jejuni* Δ*vidA* Δ*vidB* swimming in MH broth with methylcellulose (40 cP).

**Movie S7. Flagellar motility of WT *C. jejuni* overexpressing VidB in low-viscosity media.** Dark-field microscopy of WT *C. jejuni* containing a plasmid overexpressing VidB swimming in MH broth alone (1 cP).

**Movie S8. Flagellar motility of WT *C. jejuni* overexpressing VidB in high-viscosity media.** Dark-field microscopy of WT *C. jejuni* containing a plasmid overexpressing VidB swimming in MH broth with methylcellulose (40 cP).
